# Geological and Climatic Factors Affect the Population Genetic Connectivity in *Mirabilis himalaica* (Nyctaginaceae): Insight From Phylogeography and Dispersal Corridors in the Himalaya-Hengduan Biodiversity Hotspot

**DOI:** 10.3389/fpls.2019.01721

**Published:** 2020-01-31

**Authors:** Hum Kala Rana, Dong Luo, Santosh Kumar Rana, Hang Sun

**Affiliations:** ^1^ Key Laboratory for Plant Diversity and Biogeography of East Asia, Kunming Institute of Botany, CAS, Kunming, China; ^2^ University of Chinese Academy of Sciences, Beijing, China

**Keywords:** climate change, dispersal corridors, ensemble species distribution modelling, genetic connectivity, *Mirabilis himalaica*, phylogeography

## Abstract

The genetic architecture within a species in the Himalaya-Hengduan Mountains (HHM) region was considered as the consolidated consequence of historical orogenesis and climatic oscillations. The visualization of dispersal corridors as the function of population genetic connectivity became crucial to elucidate the spatiotemporal dynamics of organisms. However, geodiversity and physical barriers created by paleo geo-climatic events acted vigorously to impact notable alterations in the phylogeographic pattern and dispersal corridors. Therefore, to achieve detailed phylogeography, locate dispersal corridors and estimate genetic connectivity, we integrated phylogeography with species distribution modelling and least cost path of *Mirabilis himalaica* (Edgew.) Heimerl in the HHM. We amplified four cpDNA regions (*pet*L-*psb*E, *rps*16-*trn*K, *rps*16 intron, *trn*S-*trn*G), and a low copy nuclear gene (*G3pdh*) from 241 individuals of 29 populations. SAMOVA, genealogical relationships, and phylogenetic analysis revealed four spatially structured phylogroups for *M. himalaica* with the onset of diversification in late Pliocene (c. 3.64 Ma). No recent demographic growth was supported by results of neutrality tests, mismatch distribution analysis and Bayesian skyline plot. Paleo-distribution modelling revealed the range dynamics of *M. himalaica* to be highly sensitive to geo-climatic change with limited long-distance dispersal ability and potential evolutionary adaptation. Furthermore, river drainage systems, valleys and mountain gorges were identified as the corridors for population genetic connectivity among the populations. It is concluded that recent intense mountain uplift and subsequent climatic alterations including monsoonal changes since Pliocene or early Pleistocene formulated fragmented habitats and diverse ecology that governed the habitat connectivity, evolutionary and demographic history of *M. himalaica.* The integrative genetic and geospatial method would bring new implications for the evolutionary process and conservation priority of HHM endemic species.

## Introduction

The Himalaya and the Hengduan Mountains (HHM) are one of the main biodiversity hotspots of the Northern Hemisphere formed as a result of the collision between the Indian and Eurasian plate, started c. 55–50 million years ago (Ma) (Yin and Harrison, 2000; Dupont-Nivet et al., 2010). Mulch and Chamberlain (2006) suggests that the orogeny of the Hengduan Mountains occurred as a final propagation of the uplift after late Miocene (c. 10 Ma). In addition, recent violent Qinghai-Tibetan Plateau (QTP) uplifts (c. 3.6–1.5 Ma), and associated intensification of the Asian monsoon (c. 2.6–3.6 Ma) caused dramatic changes in the biodiversity of HHM and QTP regions alongside alterations in regional landforms and environmental heterogeneity (Li et al., 1979; Li and Fang, 1999; An et al., 2001).

The incredible biodiversity and genetic architecture in the HHM region were considered as the aftereffect of historical orogenesis independently or in combination with climatic oscillations (Avise, 2009; Favre et al., 2015). Moreover, the geological and climatic factors enormously influence the spatiotemporal pattern of temperate plant species, including historical dispersal and gene flow among the populations (Yu et al., 2015). Such orogenic uplift of mountains results in the formation of the geographical barriers, leading to fragmented habitat, loss of dispersal corridors, and restricted gene flow among populations; such factors furnish circumstances for divergence owing to genetic drift and natural selection (Liu et al., 2006; Meng et al., 2017). Historical dispersal process is likely to be a key factor to determine the current spatial population structure of species affected by the geological and climatic alterations (Hewitt, 2000). Therefore, it is crucial to locate historical dispersal for a better understanding of the demographic and evolutionary history of species, as an outcome of paleo geo-climatic events (Brown and Yoder, 2015).

Phylogeographic approach integrated with ensemble species distribution modelling (SDM) analysis provides comprehension of how paleo-environmental alterations in landscape and climate have governed species distributions and demographical history (Avise, 2000; Wang and Yan, 2014). In addition, landscape genetics has been widely used for modelling the dispersal corridors of species (Wang et al., 2009; Yu et al., 2017). Least cost path (LCP) uses landscape genetic approach coupled with species distribution models and population genetic data to recognize population genetic connectivity in a spatially explicit framework (Chan et al., 2011; Yu et al., 2015). LCP contributes to the most suitable habitat and fewest movement barriers providing the best theoretical route for dispersal in organisms (Larkin et al., 2004).

Over the last decade, a number of phylogeographic studies have investigated the link between organismic evolution of plant species and the geologic uplift associated with climate changes in the HHM and QTP (Qiu et al., 2011; Jian et al., 2015; Luo et al., 2016). For instance, phylogeographic studies on *Primula tibetica* related to the effects of Quaternary climatic oscillations in QTP (Ren et al., 2017a), *Metrocoris sichuanensis* concerned with geological effects in Sichuan Basin (Ye et al., 2018), and *Allium* section *Sikkimensia* linked with Hengduan Mountains massif uplift (Xie et al., 2019). They have demonstrated that the dramatic uplift independently or in combination with Pleistocene glaciations influenced their patterns of genetic variation. However, few studies have compared the relative significance of geo-climatic mechanisms influencing the historical dispersal and the population genetic connectivity in this region (Rana et al., 2019).

The present study focuses on the HHM endemic *Mirabilis himalaica* (Nyctaginaceae) that distributes from the Western Himalaya to the Hengduan Mountains that stretch from N India, WC Nepal, Bhutan, and S Xizang, SW Gansu, N Sichuan, NW Yunnan in the HHM region (Lu et al., 2003; Wang et al., 2019). The genus *Mirabilis* L. constitutes ~60 spp., predominantly in temperate and tropical North America and South America and single species in Asia (*M. himalaica*; Lu et al., 2003; Spellenberg, 2003; Wang et al., 2019). *Mirabilis himalaica* grows in thickets, grasslands, dry and warm river valleys between 1,400–3,800 m (Lu et al., 2003; Wang et al., 2019). For the present work, we hypothesize that the geological events to be the most important driver, if the divergence occurs before mid-Pleistocene with relatively old divergence time. We further presumed that the Pleistocene climate along with geological events greatly influenced the lineage colonization and spatiotemporal distribution of the *M. himalaica*. Therefore, we set the following primary goals for our research: 1) to identify genetic diversity and phylogeographic structure; 2) to date the divergence time between lineages and locate population genetic connectivity during the late Quaternary in the HHM region; 3) and to elucidate how paleo geological change and climatic oscillations have affected comprehensive evolutionary and demographic history of *M. himalaica*. This study represents the integrative approach of maternally inherited (cpDNA) and biparentally inherited (*G3pdh*) population genetic data in combination with the SDM and LCP approach throughout the distribution range of species to outreach the role played by the historical processes on the present-day spatial population structure of organisms in the HHM.

## Materials and Methods

### Sampling, DNA Extraction, PCR Amplification, and Sequencing

**Figure 1:**
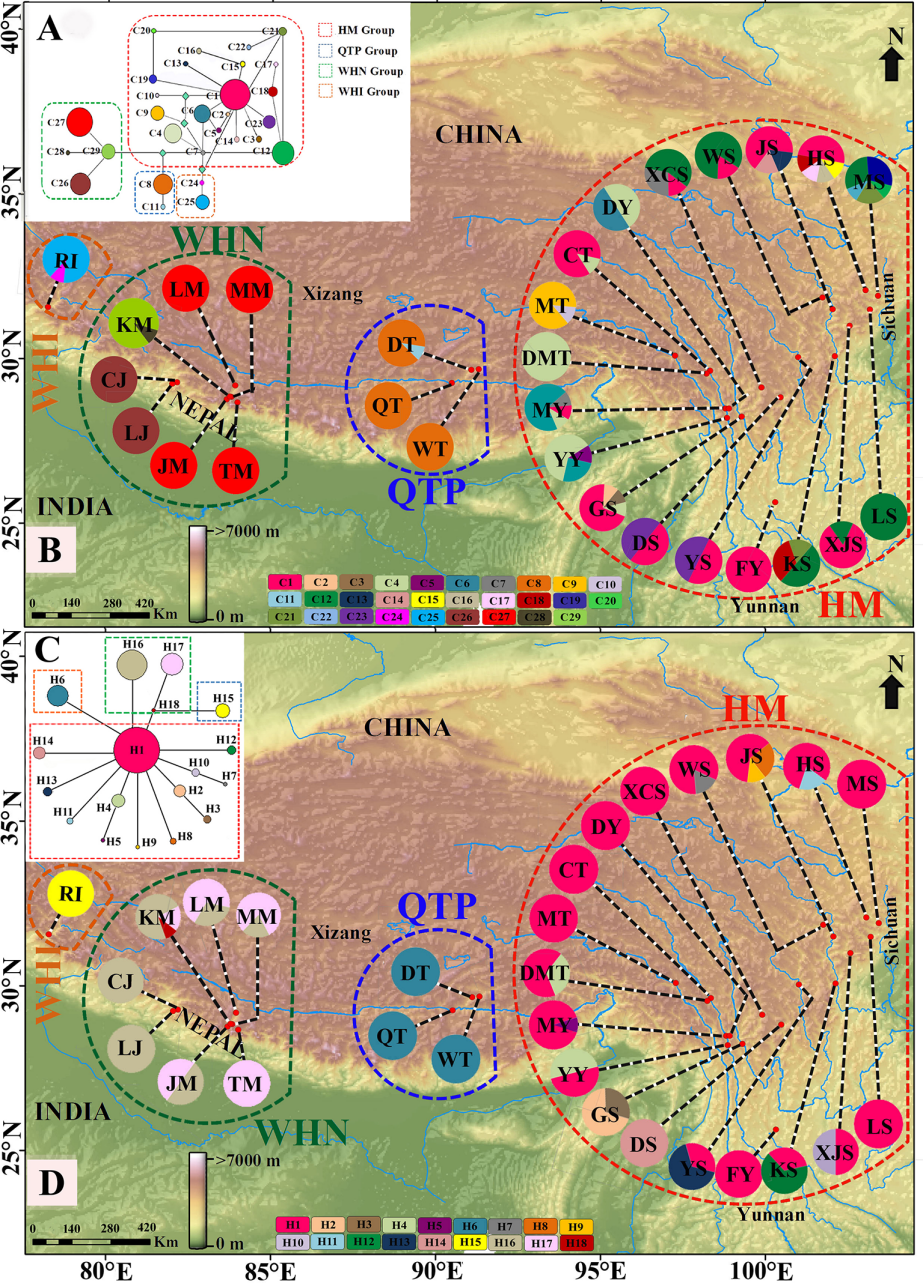
The MP median-joining networks of 29 chlorotypes (C1–C29) of cpDNA marker **(A)**/18 haplotypes (H1–H18) of *G3pdh* marker **(C)** and geographic locations of 29 populations of *M. himalaica* along with the distribution of the 29 chlorotypes of cpDNA **(B)**/18 haplotypes of *G3pdh* **(D)** detected among them (see **Table 1** for population codes). In network figure, the size of circles corresponds to the frequency of each chloro/haplotypes and the light green squared box represents chloro/haplotypes missing from the dataset and each branch represents one mutation. Pie charts indicate the sample of each population and divisions within correspond to chloro/haplotypes with a number of individuals. The dotted line delineates the phylogroups (HM, Hengduan Mountains group; QTP, Qinghai-Tibetan Plateau group; WHN, Western Himalaya Nepal group; and WHI, Western Himalaya India group). Map source: http://www.diva‐gis.org/ and http://www.worldclim.org.

In total, 241 individuals, from 29 populations covering the possible geographical range i.e. from the Western Himalaya to the Hengduan Mountains were sampled for the phylogeographic study of *Mirabilis himalaica* (**Figure 1** and **Supplementary Table S1**). The fresh sampled leaves of 6–10 individuals from each population (**Table 1**) were at least 30 m distance apart and dried in silica gel. Voucher specimens were deposited at National Herbarium and Plant Laboratories (KATH), Nepal, and Kunming Institute of Botany (KUN), China. Total genomic DNA was extracted from c. 30 mg silica dried leaves using a DNAsecure Plant Kit (Tiangen Biotechnology Co. Ltd., Beijing, China) following the manufacturer's protocol. After preliminary screening, we amplified four cpDNA regions i.e. petL-*psb*E*, rps*16-*trn*K, *rps*16 *intron,* and *trn*S-*trn*G, and a low copy nuclear gene i.e. *G3pdh* for each individual. PCRs were conducted in a 30 µl containing 2 µl DNA template (10–50 ng/µl), 15 µl 2x Taq Plus Master Mix with dye (Tiangen Biotech.), 1 µl 10 µM of each primer (see **Supplementary Methods S1** for detail).

**Table 1:** Haplotype diversity and nucleotide diversity within populations of *M. himalaica* based on cpDNA and low copy nuclear gene (*G3pdh*) sequence.

Population	N	Chlorotypes/Haplotypes (N)		cpDNA		*G3pdh*	
ID		cpDNA	*G3pdh*	*H*_d_	*π*	*H*_d_	*π*
**HM**							
FY	8	C1 (8)	H1(8)	0	0	0	0
GS	10	C1(7), **C2**(1), **C3**(1), C4(1)	**H2**(7), **H3**(3)	0.378	0.0003	0.467	0.0005
YY	8	C4(5), **C5**(1), C6(2)	H1(1), H4(1), H1–H4(6)	0.536	0.0003	0.538	0.0006
MY	10	C1(1), C4(1), C6(7), **C7**(1)	H1(9), **H5**(1)	0.533	0.0002	0.2	0.0004
CT	8	C1(7), C4(1)	H1(8)	0.25	0.0002	0	0
DMT	6	C4(6)	H1(4), H4(2)	0	0	0.533	0.0006
MT	8	**C9**(7), **C10**(1)	H1(8)	0.25	0.0002	0	0
WS	6	C1(1), C12(5)	H1(5), **H7**(1)	0.333	0.0003	0.333	0.0007
JS	6	C1(4), **C13**(1), **C14**(1)	H1(3), H1–**H8**(2), **H9**(1)	0.6	0.0002	0.607	0.0007
XJS	6	C1(5), C12(1)	H1(3), **H10**(3)	0.333	0.0003	0.6	0.0006
LS	8	C12(8)	H1(8)	0	0	0	0
HS	8	C1(4), **C15**(1), **C16**(1), **C17**(1), C18(1)	H1(6), H1– **H11**(2)	0.786	0.0007	0.356	0.0008
MS	10	C12(3), **C19**(3), **C20**(1), C21(2), **C22**(1)	H1(10)	0.6	0.0006	0	0
KS	6	C12(3), C18(2), C21(1)	H1(2), **H12**(4)	0.533	0.0002	0.533	0.0006
YS	6	C1(3), C23(3)	H1(2), **H13**(4)	0	0	0.533	0.0006
DS	8	C1(4), C23(4)	**H14**(8)	0	0	0	0
XCS	7	C1(1), C6(2), C12(4)	H1(7)	0.667	0.0004	0	0
DY	10	C4(5), C6(5)	H1(10)	0.556	0.0003	0	0
**QTP**							
QT	6	C8(6)	H6(6)	0	0	0	0
WT	10	C8(9), **C11**(1)	H6(10)	0	0	0	0
DT	6	C8(6)	H6(6)	0	0	0	0
**WHN**							
CJ	10	C26(10)	H16(10)	0	0	0.182	0.0002
LJ	10	C26(10)	H16(10)	0	0	0	0
JM	10	C27(10)	H16(5), H17(5)	0	0	0.556	0.0024
MM	10	C27(10)	H16(8), H17(4)	0	0	0.356	0.0015
KM	10	**C28**(1), **C29**(9)	H16(7), H17(2), H16–**H18**(1)	0.2	0.00006	0.473	0.0016
LM	10	C27(10)	H16(3), H17(7)	0	0	0.467	0.002
TM	10	C27(10)	H17(10)	0	0	0	0
**WHI**							
RI	10	**C24**(1), **C25**(9)	**H15**(10)	0.2	0.00006	0	0
**Total**	241			0.901	0.0013	0.777	0.0017

Private chlorotypes/haplotypes are given in bold print.

N, number of individuals; H_d_, haplotype diversity; *π*, nucleotide diversity.

The sequences of four cpDNA regions were concatenated, revised manually and aligned in Geneious 7.0.2 (Kearse et al., 2012). Chromatograms of the G3pdh with “double peaks” at polymorphic sites were further analyzed by inferring the identity of haplotypes within a heterogeneous individual through haplotype subtraction (Clark, 1990; Christe et al., 2014) using PHASE algorithm in DnaSP 5.0 (Librado and Rozas, 2009). The unique cpDNA/G3pdh haplotypes within populations were identified using DnaSP 5.0 by considering the polymorphic sites only. Newly generated haplotype sequence data have been deposited in GenBank (petL-*psb*E: MK792906–MK792916; *rps*16-*trn*K: MK792917–MK792925; *trn*S-*trn*G: MK792926–MK792935; *rps*16 *intron*: MK803108–MK803115; *G3pdh*: MK803090–MK803107).

### Genetic Polymorphism and Structure Analyses

Unique chlorotypes of cpDNA and haplotypes of *G3pdh* were determined in DnaSP 5.0 (Librado and Rozas, 2009). Geographical distributions of chlorotypes/haplotypes were plotted using ArcGIS 10.2.1 (ESRI, Inc., Redlands, CA, USA). To quantify the level of genetic variation, total haplotype diversity (*H*_T_) and average within-population diversity (*H*_S_) (Nei, 1975) were calculated. *G*_ST_ and *N*_ST_ were used to estimate differentiation between populations (Nei, 1975). *N*_ST_ was compared to *G*_ST_ using *U*-statistics; an observed value of *N*_ST_ > *G*_ST_ generally indicates the presence of phylogeographical structure (Pons and Petit, 1996). We computed all these parameters employing Haplonst (Pons and Petit, 1996). DnaSP was used to analyze the genetic diversity parameters, including the haplotype diversity (*H*_d_; Nei and Tajima, 1981) and nucleotide diversity (*π*; Nei and Li, 1979).

Spatial analysis of molecular variance (Samova v2.0; Dupanloup et al., 2002), was implemented to define the number of groups of populations (*K*). We also performed an analysis of molecular variance (AMOVA) to examine the genetic variation using Arlequin v3.5 (Excoffier and Lischer, 2010). The *F*-statistic (*F*_ST_/*F*_SC_/*F*_CT_) was calculated, and significance was tested for overall as well as regional populations. “Isolation by distance” (IBD) was evaluated by regressing the net nucleotide divergence between populations (*D*_A_) in contrary to their geographical distance, applying Mantel's (1967) test with 999 permutations in GenAlEx 6.5 (Peakall and Smouse, 2012). Network v5.0.0 (Bandelt et al., 1999; http://www.fluxus-engineering.com) was employed to construct median-joining networks of the cpDNA and *G3pdh* sequences to assess their genealogical relationships. All variable sites were included and weighted equally.

### Phylogenetic Analyses and Divergence Dating

**Figure 2:**
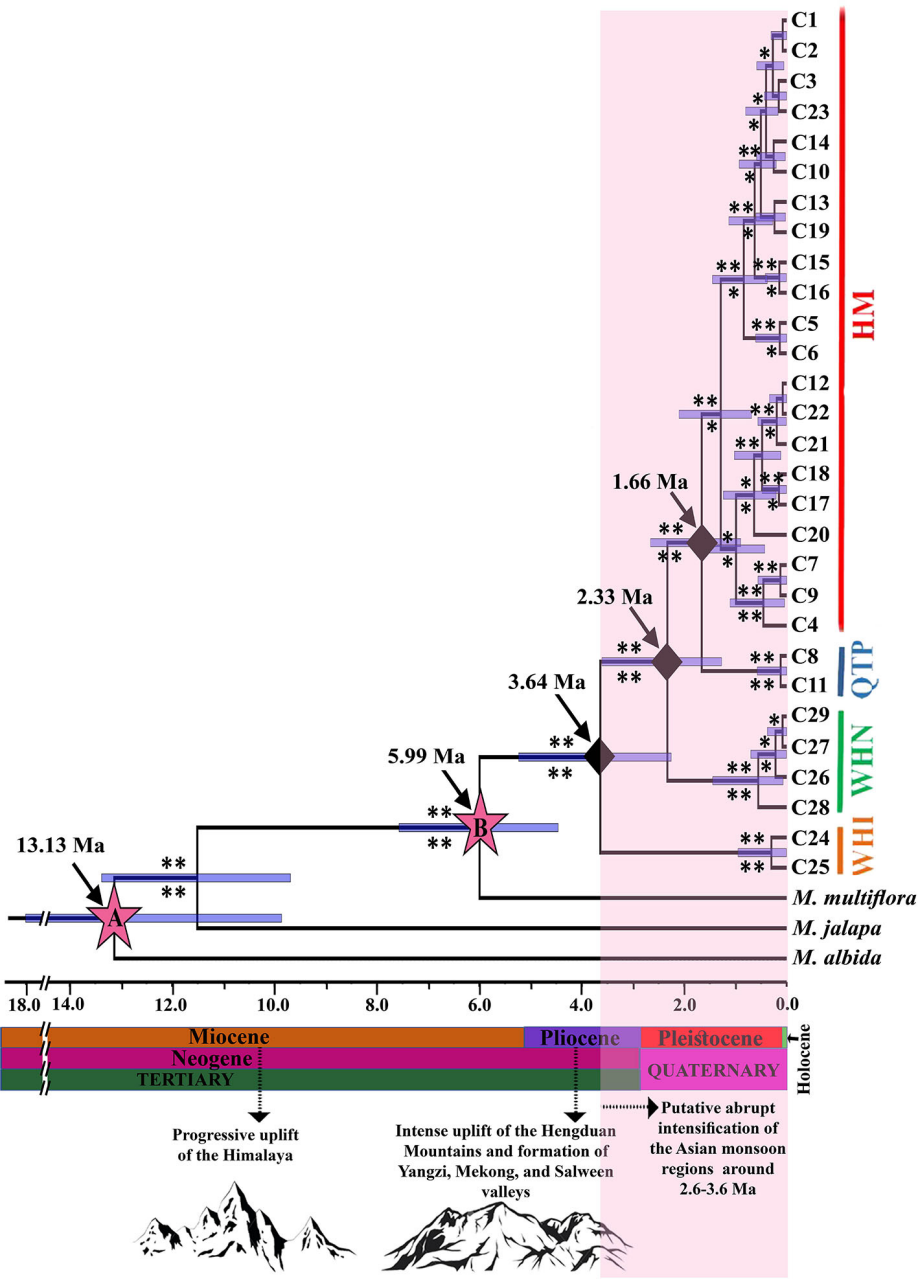
Maximum credibility clade (MCC) tree with estimated divergence times (Ma; Million Years ago) and ancestral area reconstruction of *M. himalaica* estimated using Beast analysis. Asterisks indicate posterior probabilities based on Beast (above the branches) and Bayesian analysis (below the branches). A single asterisk indicates weak or moderate support (0.5 < posterior probabilities < 0.95); two asterisks indicate strong support (posterior probabilities > 0.95). Node A and B represent the secondary calibration points based on Wang et al. (2019). The geological time scale with historical events is shown at the bottom of the phylogenetic tree.

Phylogenetic relationships among chlorotypes of cpDNA along with sequences of three closest species from *Mirabilis* L. (*M. jalapa*, EF079612; *M. multiflora*, EF079603; *M. albida*, EF079602/KR014118) as outgroups were constructed by Bayesian inference (BI) in MrBayes v3.2 (Ronquist et al., 2012) and Beast v1.8.4 (Drummond and Rambaut, 2007; Drummond et al., 2012) under the GTR+I nucleotide substitution model selected by Akaike information criterion (AIC) in jModelTest v2.1.6 (Guindon and Gascuel, 2003; Posada and Buckley, 2004) (see **Supplementary Methods S1** for detail parameters). Beast v1.8.4 was employed to estimate the temporal intraspecific divergence times (crown ages) of chlorotypes/haplotypes. We assumed a strict clock (*P =* 0.85; i.e. *P* > 0.05), based on a likelihood-ratio test (Felsenstein, 1988) in Paup* 4.0b10 (Swofford, 2002) and constant population size for the coalescent tree prior to the distribution of divergence times. We used two secondary calibration points (**Figure 2**) to estimate the lineage divergence times based on Wang et al., 2019 (A: 13.13 ± 4.2 Ma; 95% highest posterior density [HPD] intervals: 6.91–20.62 Ma; i.e. a crown age for *Mirabilis,* and B: 5.22 ± 1.7 Ma; 95% 2.53–8.18 Ma i.e. divergence time of *M. himalaica* from its North American counterparts; **Figure 2**) (see **Supplementary Methods S1** for detail parameters). Three independent runs of 20 million generations were carried out, with number of chains = 4, and sampling every 1,000 generations, where first 20% of the trees were discarded as burn-in. TreeAnnotator v1.8.4 (Drummond and Rambaut, 2007; Drummond et al., 2012) was used to obtain a maximum-credibility tree and FigTree v1.4.0 (Rambaut, 2012) was employed to view the resulting tree.

### Historical Demographic Analyses

Tajima's (1989) *D* and Fu's (1997) *F*_S_ of neutrality tests were performed using Arlequin v3.5 (Excoffier and Lischer, 2010), with 1,000 simulated samples. Pairwise mismatch distribution analysis (MDA; Rogers and Harpending, 1992) was performed in Arlequin v3.5 to detect demographic expansion events of *M. himalaica*. The MDA compared the observed frequency distribution of pairwise differences among haplotypes with their theoretical distribution expected under the ‘pure population growth’ and ‘spatial expansion’ models, respectively. With the sum of squared deviations (SSD) and Harpending's (1994) raggedness index (*H_Rag_*) ‘goodness of fit’ was tested, using 1,000 parametric bootstrap replicates.

In addition, Bayesian skyline plot (BSP: Drummond et al., 2005) generated in Beast v1.8.4 was used to reconstruct the effective population size fluctuations since the time of the most recent common ancestor for each marker and the combined markers (cpDNA and *G3pdh*). The MCMC chains (nchains = 4) were run for 20 million generations sampling every 100 generations, with effective sample size (ESS) greater than 200. We used the same settings for three independent runs to ensure the consistency of the results. The demographic history through time was reconstructed using TRACER v1.7.1 (Rambaut et al., 2018).

### Ensemble Species Distribution Modelling and Visualizing Dispersal Corridors

An ensemble of 'species distribution modelling (SDM; Guisan and Zimmermann, 2000) was carried out using “*Biomod 2*” (Thuiller et al., 2014) package implemented in R-programming language (R v3.4.1; R Development Core Team, 2016) for past (Last Interglacial, LIG; 120–140 Ka and Last Glacial Maximum, LGM; c. 22 Ka) (Otto-Bliesner et al., 2006), current (1990–2000) and future (2070; RCP 4.5). The model used 72 spatially filtered occurrence points to prevent from spatial autocorrelation that were checked in the 4-min grid (Rana et al., 2019). The eight predictive bioclimatic variables were selected based on iterative calculations of Variance Inflation Factors (VIF < 10; Fox and Weisberg, 2011) (**Table 2**), Pearson correlation (r < 0.8; **Supplementary Table S2**), followed by confirmatory test Principal component analysis (PCA) analysis implemented in R-Programming (**Supplementary Table S3**). The predictive performances of the 10 simulated models were calibrated and evaluated using 25% of the data that uses AUC (Area under ROC curve) > 0.8, TSS (True Skill Statistics); Cohen's Kappa > 0.7 (**Supplementary Table S4**). These models were then projected onto plaeo and future (2070, RCP4.5) climatic scenarios based on different General Circulation Models (one LIG, two each for LGM and future) to determine the distribution range shifts and suitable habitats of *M. himalaica*. The ensemble consensus model was converted into binary presence (1)/absence (0) applying thresholds that allow a maximum of 50% probability of the suitable habitat (Forester et al., 2013). We also reclassified changes in LIG, LGM, and future conditions compared to current suitability into retracted, stable, and expanded areas. (see **Supplementary Methods S1** for details).

**Table 2:** Variance Inflation Factor (VIF) in different test runs for the selection of explanatory variables and the correlation between predictor variables.

Variables*	Run1	Run2	Run3	Run4	Run5	Run6	Run7	Run8	Run9	Run10	Run11	Run12	Run13	Final
**Bio3**	266.92	262.67	261.07	165.58	122.17	114.85	114.85	107.59	72.92	55.25	2.09	1.58	1.28	1.28
**Bio5**	101564400	617.24	412.9	352.9	352.74	351.44	85.04	73.22	68.05	13.28	13.14	11.99	1.79	1.79
**Bio18**	253.59	248.6	236.05	224.45	173.99	170.87	125.63	120.24	27.74	16.98	16.92	3.93	3.29	3.29
**Bio15**	34.15	30.75	28.06	23.63	22.88	19.72	19.14	19.07	4.91	4.91	4.52	3.48	3.41	3.41
**Bio2**	586.31	564.37	525.05	390.21	97.15	91.81	86.95	78.46	56.54	37.57	4.68	4.43	3.44	3.44
**Bio17**	402.26	401.15	399.67	355.49	321.25	309.95	255.08	18.08	18.01	17.97	15.54	8.11	6.46	6.46
**Bio 14**	68	67.98	58.29	49.29	46.71	46.44	29.56	16.19	11.14	7.85	7.58	7.58	7.5	7.50
Bio 8	44.58	43.92	41.22	40.95	39.26	38.95	37.6	20.19	16.77	16.77	16.54	**15.15**		
Bio 13	206.01	204.29	200.85	200.76	179.01	65.68	65.66	62.52	32.95	25.94	**25.2**			
**Bio 4**	2060.64	2060.33	802.28	184.45	122.09	120.19	103.06	98.86	75.07	**74.06**				
Bio 9	227.01	226.28	224.67	203.95	193.99	193.68	184.71	102.4	**102.29**					
Bio 12	507.76	484.28	478.02	398.16	378.53	286.9	254.81	**253.6**						
Bio 19	299.27	295.97	295.96	295.57	276.03	273.63	**259.23**							
Bio 1	6500.56	6400.72	4109.3	392.65	390.3	**390.3**								
Bio 16	518.08	515.93	509.47	497.88	**453.91**									
Bio 7	87706640	992.46	604.94	**599.07**										
Bio 10	8658.9	8658.5	**5173.99**											
Bio 11	18636.34	**18494.23**												
Bio 6	**180778400**													

The bold text variables were selected as a subset of explanatory variables for the model after excluding variables with VIF > 10 in each subsequent test run. In addition, to the final subset Bio4 is also considered for model building from Pearson's correlation analysis.

*See **Supplementary Table S7** for an abbreviated form of 19 bioclimatic variables.

The dispersal corridors were identified following the least cost path (LCP) methods using SDMtoolbox v2.0 (Brown et al., 2017) under the four climatic scenarios LIG, LGM, current, and future. For this analysis, we adopted the haplotype information of 29 localities, and populations that share haplotypes from two molecular markers (cpDNA and *G3pdh*) (**Figures 1B**, **D**). Firstly, we obtained a dispersal cost layer (resistance layer) by inverting the SDMs, and subsequently, we created a cost distance raster for each sampling locality using the resistance layer. Corridor layers were established based on the cost distance raster between two localities that shared haplotypes. To avoid oversimplifying landscape processes, we classified the value of each corridor layer into four intervals (three cutoff values: 1%, 2%, 5%) and reclassified as new values (5, 2, 1, 0, respectively). Finally, we summed up and standardized all of the pairwise reclassified corridor layers and identified dispersal maps of *M. himalaica* in an explicit landscape.

## Results

### Haplotype Diversity and Distributions

The total aligned sequences of the four combined cpDNA regions are 3290 bp (petL-*psb*E/838 bp*, rps*16-*trn*K/602 bp, *rps*16 *intron*/883 bp*, trn*S-*trn*G/967 bp) with 28 polymorphic sites and 71 indels (**Supplementary Table S5**). In total, 29 chlorotypes (C1–C29) were identified among 241 individuals (**Table 1** and **Figure 1B**). The most common chlorotype was C1 (shared by 11 populations) followed by C12 (shared by six populations) (**Table 1** and **Figure 1B**). Out of total chlorotypes, WHI and QTP groups (grouping by Samova) harboured two chlorotypes each (6.9%), WHN group contained four chlorotypes (13.8%), and the remaining 21 were occupied by HM group (72.4%); indicating a very high level of molecular diversity in HM region. Nineteen chlorotypes (65.5% of the total) were relatively rare, each of them restricted to a single population, whereas 15 of them were distributed in the HM region.

Out of 241 individuals, only about 5% (11 individuals) are heterozygous for partial *G3pdh* sequence under haplotypes H1, H4, H8, H11, H16, and H18. Population YY is the most diverse with six heterozygous individuals. The length of the aligned sequence of *G3pdh* was 941 bp, containing 18 nuclear haplotypes with 20 polymorphic sites (**Supplementary Table S6**). Thirteen haplotypes (72.2%) were unique, each endemic to a single population; 11 of them (84.6%) were restricted to the HM region. H1 was the most common haplotype within 16 populations of HM regions (**Figure 1C**). The chlorotype/haplotype frequencies and distributions in populations are listed and shown in **Table 1** and **Figure 1**.

### Genetic Polymorphism and Population Structure

Total haplotype diversity (*H*_d_)/nucleotide diversity (*π*) for the cpDNA and *G3pdh* sequences were 0.901/0.0013 and 0.777/0.00173, respectively. For cpDNA, the highest value of *H*_d_ and *π* falls in population HS (*H*_d_ = 0.786; *π =* 0.0006); whereas for *G3pdh*, population JS exhibited particularly a high value (*H*_d_ = 0.607; *π =* 0.0007) (**Table 1**). Total genetic diversity (*H*_T_) in the overall population of *M. himalaica* were 0.917/0.803 (cpDNA/*G3pdh*) (**Table 3**). The average within-population diversity for both cpDNA/*G3pdh* (*H*_S_ = 0.303/0.340) was also relatively high. Both, *H*_T_ and *H*_S_ are highest in the HM group (for WHI group not calculated, because of only one population). The inter-population differentiation (*G*_ST_) of the two datasets i.e. cpDNA and *G3pdh* was 0.669 and 0.577, respectively. For both datasets, *N*_ST_ was significantly greater than *G*_ST_ (*U* > 1.96, *p <* 0.05), indicating the existing phylogeographical structure in *M. himalaica* (**Table 3**).

**Table 3:** Estimates of Genetic diversity and genetic differentiation of *M. himalaica* using cpDNA and *G3pdh* sequences.

Sequence data	n	*H*_S_	*H*_T_	*G*_ST_	*N*_ST_†
cpDNA	29	0.303(0.0544)	0.917 (0.0179)	0.669 (0.0598)	0.902 (0.0278) *
*G3pdh*	18	0.340 (0.0611)	0.803 (0.0609)	0.577 (0.0662)	0.629 (0.0672) ^NS^

n, Number of Haplotypes; *H*_S_, Mean genetic diversity within populations; *H*_T_, Total genetic diversity; *G*_ST_, Genetic differentiation use only of the allelic frequencies; *N*_ST_, Genetic differentiation considered similarities between the chlorotypes/haplotypes; NS, Not significant.

*N_ST_ is significantly different from *G*_ST_ (U > 1.96; P < 0.05).

† = Permutation test (P) with *G*_ST_.

Samova of cpDNA reached a platform when *K =* 5 (*F*_CT_ = 0.872) and revealed a substantial spatial population genetic structure with five groups, i.e. HM (Hengduan Mountain) group with 18 populations, QTP (Qinghai-Tibetan Plateau) group with three populations, WHN (Western Himalaya Nepal: WC Nepal) I group with three populations (CJ, LJ, KM), WHN II group with four populations (MM, JM, LM, TM) and WHI (Western Himalaya India: Himachal Pradesh, India) group with one population (**Supplementary Figure S1**). Samova of *G3pdh* data reached a platform when *K =* 6 (*F*_CT__=_ 0.6996, **Supplementary Figure S1**) and divided two same groups (QTP and WHI) like that of the cpDNA dataset. While the WHN was separated into two different subgroups (WHN1: CJ, LJ, MM, KM; WHN2: JM, LM, TM), and the HM was also divided into two subgroups (GS vs. other 17 populations). Considering the geographical unit of the HHM and the unstable population MM, we merged the divided subgroups and regarded four structured phylogroups (HM, QTP, WHN, and WHI; **Figure 1**) for *M. himalaica*. This grouping is also consistent with the Samova grouping (*K* = 4) of cpDNA. Thus, based on four phylogroups, there is greater genetic partition among groups (78.58%/52.92%), than among populations within groups (17.30%/25.05%) or within populations (4.11%/22.03%) (**Table 4**). Examining the genealogical relationships/MJ network for chlorotypes and haplotypes, four clusters (HM, QTP, WHN, and WHI) were formed corresponding to the defined phylogroups (**Figures 1A**, **C**). The significant *F-*statistics from AMOVA suggested structured population within groups (**Table 4**). Additionally, the Mantel test revealed a statistically significant pattern of (IBD) for both cpDNA and *G3pdh* (*r*_M_ *_=_* 0.62/0.43; *P =* 0.001) (**Table 4**).

**Table 4:** Results of Analysis of molecular variance (AMOVA) of *M. himalaica* for cpDNA and low copy nuclear gene (*G3pdh*) sequence data, along with the results of the ‘isolation by distance’ analysis using Mantel tests in GenAlEx.

Source of Variation	cpDNA							*G3pdh*					
	d.f.	SS	VC	Variation %	*F*_ST_/*F*_SC_/*F*_CT_	*r*_M_		d.f.	SS	VC	Variation %	*F*_ST_/*F*_SC_/*F*_CT_	*r*_M_
Among populations	28	2205	9.42	93.97	0.94*	0.62*		28	151.89	0.60	71.73	0.72*	0.43*
Within populations	212	128	0.6	6.03				223	52.58	0.24	28.27		
Total	240	2333	10					251	204.47	0.83			
Among groups	3	1668	11.6	78.58	0.96*/0.81*/0.79*			3	88.31	0.57	52.92	0.78*/0.53*/0.53*	
Among populations within groups	25	537	2.55	17.30				25	63.59	0.27	25.05		
Within populations	212	128	0.6	4.11				223	52.58	0.24	22.03		
Total	240	2333	14.7					251	204.47	1.07			

*P < 0.01; d.f., Degrees of freedom; SS , Sum of squares; VC, Variance components; *F*_ST_/*F*_SC_/*F*_CT_, Fixation indices; *r*_M_, Mantel correlation coefficient.

### Phylogenetic Relationships and Divergence Time

The cpDNA topologies generated in MrBayes and Beast indicated *M. himalaica* was monophyletic with high posterior probabilities values which clustered the haplotypes into four lineages (**Figure 2**), corresponding to the four spatial population genetic groupings and genealogical relationships. Based on molecular dating, the onset of diversification for *M. himalaica* appeared in late Pliocene (crown age: 3.64 Ma; 95% HPD: 2.42–5.62 Ma; **Figure 2**) i.e. the first splitting event between the WHI lineage and the ancestor of the WHN, QTP, and HM lineages. Subsequently, the second splitting event between WHN lineage and the ancestor of QTP and HM lineage is 2.33 Ma (95% HPD: 1.37–3.87 Ma). Ultimately, the third splitting event between QTP and HM lineages occur during 1.66 Ma (95% HPD: 0.96–2.86 Ma).

### Historical Demography

Tajima’s *D* and Fu’s *F*s values were not significant, suggesting that *M. himalaica* conforms to the neutral hypothesis and these populations did not experience recent demographic expansion events (**Table 5**). The mismatch distribution for the overall chlorotypes/haplotypes were multimodal or bimodal (**Supplementary Figure S2**). While, the *SSD* and *H*_Rag_ statistics indicated a good statistical fit to the pure population growth and/or the spatial expansion model, excepting pure population growth in cpDNA data (p > 0.05; **Table 5**). This result suggested *M. himalaica* has experienced demographic events with structured populations that are shrinking in size or demographic equilibrium (Rogers and Harpending, 1992; Harpending, 1994).

**Table 5:** Results showing mismatch distribution analysis under models of (A) pure population growth and (B) spatial expansion, and neutrality test for the cpDNA and *G3pdh* sequence of *M. himalaica;* tests were conducted in ARLEQUIN with the sum of squared deviations (SSD) and Harpending's (1994) raggedness index (*H*_Rag_).

Sequence	Model	SSD	*P-*value	*H*_Rag_	*P-*value	Tajima's *D* test		Fu's *F_S_* test	
						*D*	*p-*value	*F*_S_	*p*-value
cpDNA	A	0.031	0.031	0.031	0	-0.22	0.449	11.25	0.981
	B	0.028	0.254	0.031	0.226				
*G3pdh*	A	0.109	0.242	0.066	0.273	-1.31	0.057	-6.75	0.027
	B	0.109	0.088	0.066	0.292				

BSP reconstructions based on combined markers (cpDNA and *G3pdh*) showed that the population of *M. himalaica* was quite stable after an ever-increasing phase during 0.6–0.075 Ma (**Supplementary Figure S3**), indicating no recent demographic expansion events. This case is similar for cpDNA marker when performed BSP reconstruction, while BSP of *G3pdh* showed a prolonged phase of demographic stability or no distinct population expansion (**Supplementary Figure S3**).

### Ensemble Species Distribution Modelling and Visualization of Dispersal Corridors

**Figure 3:**
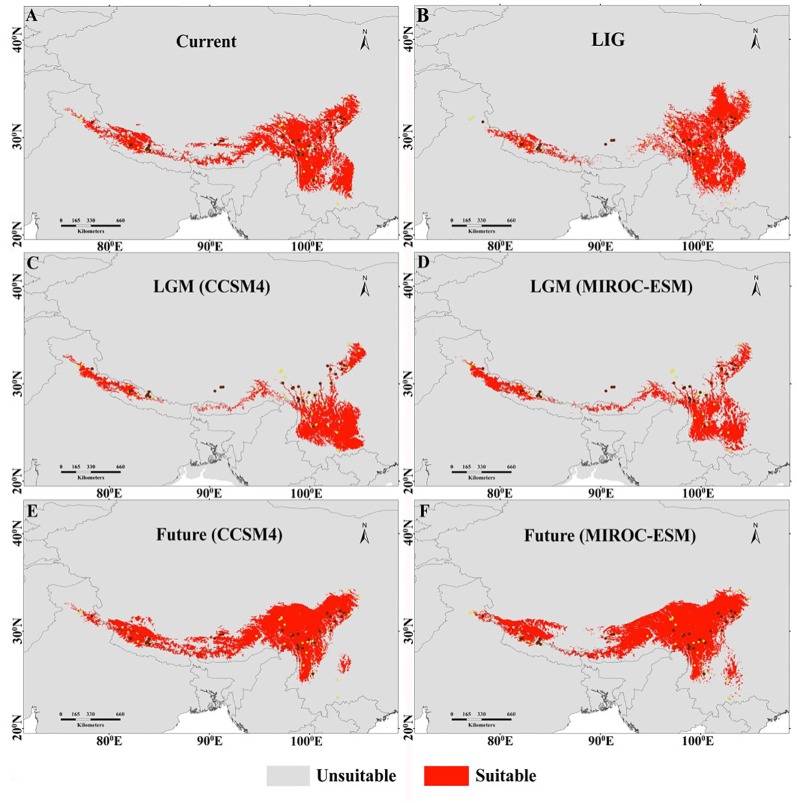
Habitat suitability models of *M. himalaica* predicted by ensemble Species Distribution Models under the assumption of the four climate scenarios: **(A)** Current (*1990–2000*); **(B)** LIG (*c. 120–140* Ka); **(C**, **D)**, LGM (*c. 22*Ka; based on the output of two GCMs; and **(E**, **F)** future (*2070;* based on the output of two GCMs under RCP4.5). Brown dots represent the location of populations in this study, and yellow dots correspond to all available occurrence locations used in species distribution modelling. The light grey regions in the map represent the area below the omission error regarded as not suitable, whereas the red regions indicate the area suitable for distribution of the species.

**Figure 4:**
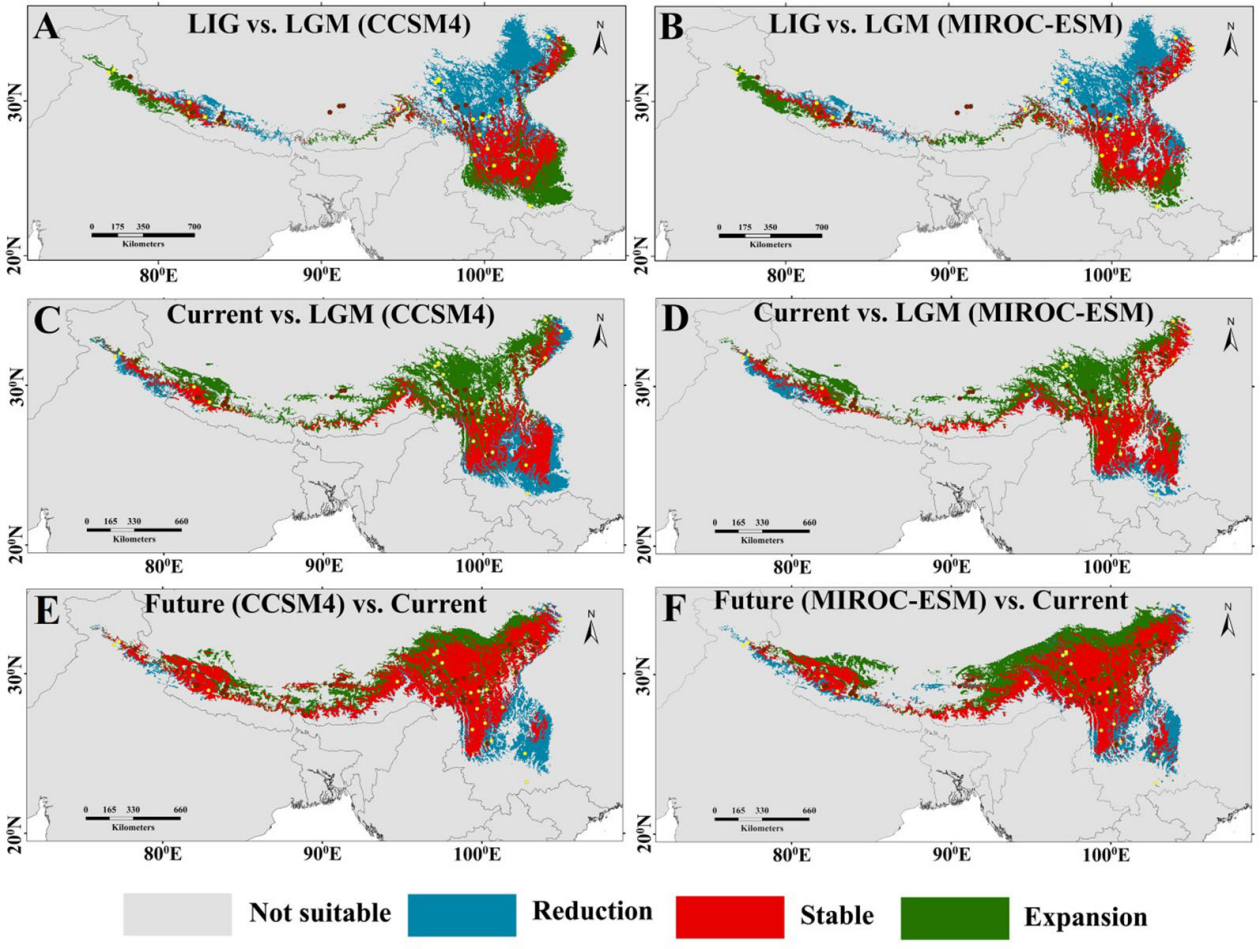
Maps of the potential Reduction, stable, and expansion areas compared between three climate scenarios: **(A)** LIG versus LGM (based on the output of CCSM4); **(B)** LIG versus LGM (based on the output of MIROC-ESM); **(C)** current versus LGM (based on the output of CCSM4); **(D)** current versus LGM (based on the output of MIROC-ESM); **(E)** future (based on the output of CCSM4, RCP4.5) versus current; versus current and **(F)** future (based on the output of MIROC-ESM, RCP4.5) versus current. See legend for representations of coloured regions.

The projected current distributions were generally the representations of the actual distributions and suitable habitat, which is consistent with present occurrence records (**Figure 3A**) except certain parts of the Eastern Himalaya. The paleodistribution reconstruction showed that during LIG, *M. himalaica* presented fragmented distribution patterns in the HM, and parts of the Western Himalaya (**Figure 3B**). Later during LGM, the species became more prominent towards the South of the HM (North Yunnan), WHI, and few in the southeastern QTP region (**Figures 3C**, **D**). The LGM distribution range of *M. himalaica* predicted using two models (CCSM4, MIROC-ESM) varies in some parts of the Western Himalaya and the HM (**Figures 3C**, **D**). In terms of the stability, we found that no matter which model was used, the distribution of *M. himalaica* during the LGM was farther south than the present one, suggesting northward expansion of populations after the LGM (**Figures 4C**, **D**). In addition, the predicted future distributions based on the MIROC-ESM model showed more migration to the north-west in the HM and west of the Himalaya compared to the present (**Figures 3F** and **4F**). The result varies from the CCSM4 model, which was showing relatively more expansion towards the west in all regions (**Figures 3E** and **4E**). It was predicted that species range expanded to southwards from LIG to LGM, but the range expansion is towards north-westwards after LGM up to current and the future (**Figures 3** and **4**).

**Figure 5:**
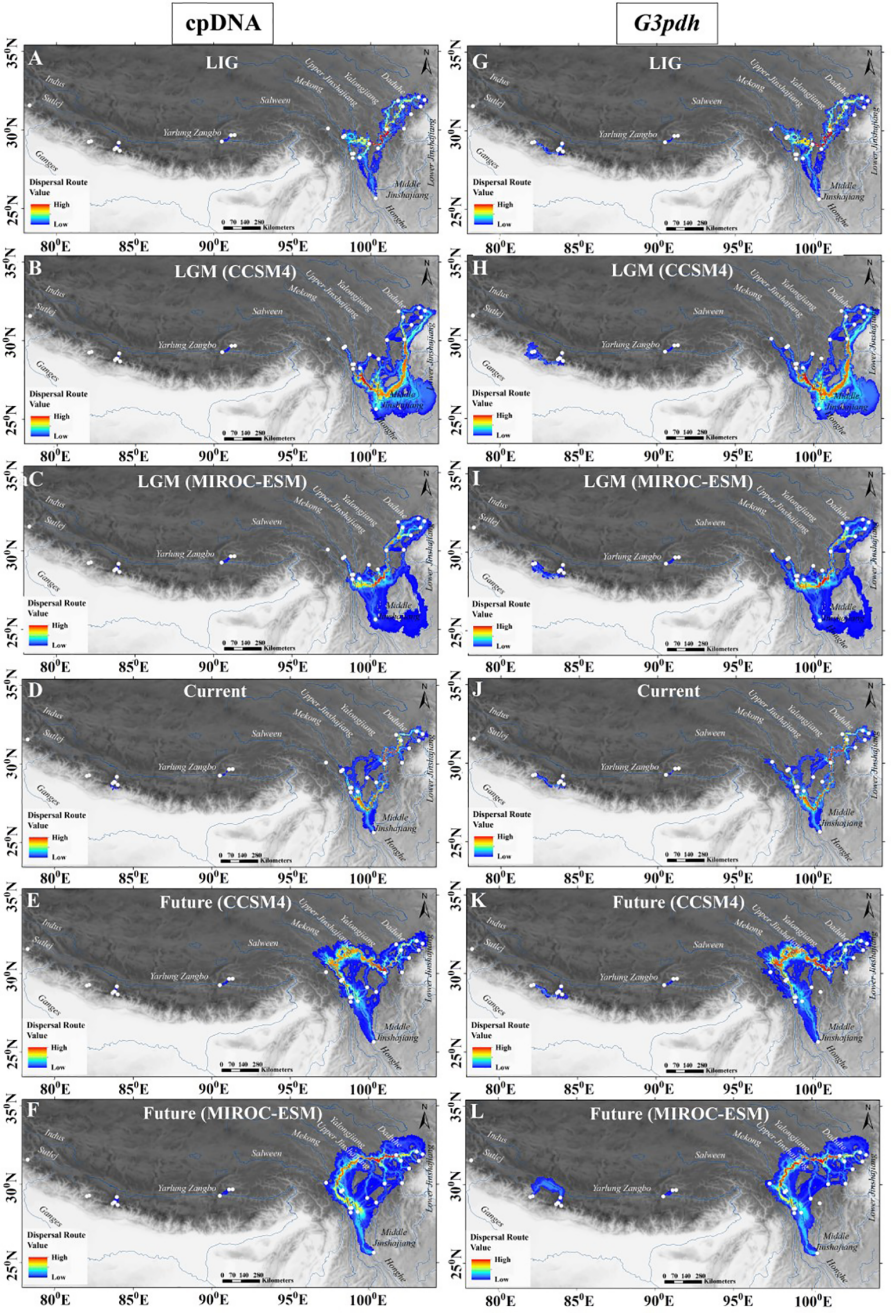
Potential dispersal corridors of *M. himalaica* in HHM based on cpDNA **(A**–**F)** and *G3pdh* **(G**–**L)** for the four periods (LIG, LGM, current, and future). Population genetic connectivity ranges from its highest values in red to its lowest values in blue.

Based on the least cost path analysis, putative dispersal corridors for the four periods (i.e. LIG, LGM, current, and future) were visualized using cpDNA and *G3pdh* markers (**Figure 5**). When comparing the dispersal routes across different time periods and genetic markers, dispersal generally followed isolated corridors between regional populations. There were no traces of connectivity between the populations of HM with the QTP and the Western Himalayas, and vice versa. This might be due to significant ridges and peaks of the local landscape in the Himalaya which formed a spatial barrier that intensified and fixed genetic isolation. The result exhibited continuous patterns of landscape connectivity among the populations present in the HM, indicating the existence of a dispersal corridor along mountains in the Hengduan regions. Besides the HM region, partial dispersal routes were also identified in the QTP and the WHN with average to low connectivity level (**Figure 5**). The cpDNA data did not show connectivity between two population groups from Jumla and Mustang–Manang of WHN; but *G3pdh* data revealed an additional area of dispersal in WHN beyond the dispersal corridor evidenced from the cpDNA data for the region (**Figure 5**). However, in this geologically dynamic region, the corridor shifts its route in different periods based on the distribution pattern, indicating that the dispersal corridor is not static for *M. himalaica* in the HHM. The distinct dispersal corridor in the middle of the HM during LIG through gorges of mountains to Yalongjiang and Daduhe rivers, shift to the south in the LGM showing strong connectivity through the middle Jinshajiang following Yalonjiang and paleo-route of Daduhe river. Similarly, in the current condition the high-value corridor passes through upper Jinshajiang, Yalongjiang, Daduhe rivers and rivulets or streams; whereas, in the future, the species is likely to lose its corridor in the south HM and shift to northwards following the spatial distribution range. In addition, a relatively larger area of dispersal was identified during the LGM period for both the makers (**Figures 5B**, **C**, **H**, **I**) compared to others. The strongest population genetic connectivity for *M. himalaica* occurred along with the river systems in the HM region.

## Discussion

### Evolutionary and Demographic History

It was speculated that the ancestor of *Mirabilis himalaica* might have migrated from North America to the Himalayas either by Beringia or through long-distance dispersal, evolving allopatrically into the extant species (Wang et al., 2019). *Mirabilis himalaica* diverged from the closest taxa during Early Pliocene c. 5.99 Ma (stem age; 95% HPD: 4.79–8.13 Ma), and the separation of the lineages spanning late Pliocene to early Pleistocene between 3.64–2.33 Ma (**Figure 2**). Late Miocene QTP uplift might have triggered speciation of *M. himalaica* in combination with the plentiful environmental alterations in the QTP and adjacent regions (Li et al., 1979). During the recent violent QTP uplifts (c. 3.6–1.5 Ma), intensification of the Asian monsoon (c. 3.6–2.6 Ma) (Li et al., 1979; An et al., 2001; Sun and Wang, 2005) were in progress, involving several mountain ranges in the HHM biodiversity hotspot (Harrison et al., 1992; Royden et al., 2008; Xie et al., 2019). Furthermore, tectonic events and climate change have set off the lineage divergence and rapidly occupying the newly available terrain during the Pleistocene. The uplift process might have coupled with the Pleistocene climatic oscillations leading to habitat diversification, restricted gene flow, and finally differentiation of the species (Xie et al., 2014; Luo et al., 2017; Shahzad et al., 2017; Ren et al., 2017a).

In addition, the glacial cycles of the Quaternary period in the HHM region have likely affected the demographic history of focal species. The results of the neutrality tests and MDA in conjunction with BSPs analysis suggested that *M. himalaica* did not experienced recent expansion events. Considering the poor ability to seed disperse, associated with the intense altitudinal gradient characterizing the Himalaya. We speculated that there is no large-scale distribution range expansion/contraction, while four lineages of *M. himalaica,* after diverging from each other survived *in situ* or experienced restricted regional migration (SDM and LCP) to shape the current phylogeographical pattern and the effective population size of all lineages remained relatively constant throughout the evolutionary period. From SDM analysis, during LGM the species was progressively confined to southwards in the HM, and traces of habitat expansion occurs in WHI, southeastern QTP region (**Figures 4C**, **D**) compared to predicted current and LIG distributions. The reason behind the southward movement of populations lies in the fact that the northernmost part of subtropical Central and Northern China was cooler at least 7–10°C and dryer by 200–300 mm/year (Sun and Chen, 1991; Zhou et al., 1991). At the time both steppe and desert vegetation expanded in a west to south directions. The localized cooling caused by glacial advance and later, subsequent warming due to glacial retreat might have exposed new habitats for colonization (Matthews, 1992) in the Northern and Western regions of the HHM. Therefore, our results suggested geological events along with climatic oscillations including monsoonal system and the Quaternary glaciation could have facilitated the formation and divergence of lineage and led to the accumulation of genetic diversity and differentiation of *M. himalaica* in the HHM region.

### Pleistocene Unstable Habitat Connectivity

In this study, we identified the possible dispersal routes of *M. himalaica* since LIG to the future conditions in the HHM. Despite the adequacy of these mountains as a barrier to dispersal (Oheimb et al., 2013), there are several gorges and passes through the Main Divide, which might have permitted the dispersal of *M. himalaica*. Consequently, the major River system and the river valleys act as a dispersal corridor or recolonization route within the structured populations. Strikingly, the connectivity of dynamic habitats for *M. himalaica* was supported by the ensemble SDM prediction, which indicated that the population habitat connectivity also experienced potent change since LIG. Further, population genetic connectivity analysis based on the genetic data and SDM resistance layer similarly indicated that population genetic connectivity was prominent from LIG to current, and later in future conditions too (**Figure 5**). Population genetic connectivity and historical demographic changes of extant organisms are often associated with cyclical Pleistocene glaciations (Hewitt, 2000); and appears to be an important driver of inconsistency in the population genetic connectivity in *M. himalaica*. Besides, Pleistocene glaciations likely were restricted to high or middle elevations and did not affect deep slopes or valleys in the south-west mountainous region of China (Qu et al., 2014). As a result, the migration of populations to the deep slopes or valleys might have formulated strong dispersal corridors in the southern Hengduan Mountains during LGM compared to LIG. Our result showed that the corridors shift northward after LGM up to the future, indicating a purely consistent pattern with the spatial distribution. However, the rapid range change during the LIG to the future transition probably contributed to the patterns of genetic connectivity of local populations.

### Response to Climate Change and Implications for Conservation

According to the forecasted future potential distribution, the focal species shift north-westward losing their potential suitability in hilly and lower mountainous regions by 2070 (**Figures 4E**, **F**). This extensive elevational shift might be due to global warming which combined with other biotic/abiotic factors fabricating unsuitable habitats at lower elevations. The annual mean warming rates during the period 1901–2020 was 0.19°C/decade (Ren et al., 2017b; Krishnan et al., 2019) over the Hindu Kush Himalaya, which is expected to increase further in future. In addition, the rate of warming for the Himalaya has been reported approximately three times (i.e. 1.5 °C/year) than the global average (i.e. 0.06 °C/year) (Shrestha et al., 2012). This increased warming rate at Himalaya region may prompt a significant issue for montane species. Likewise, the northward elevational shift of the species in the future does not ensure an increase in plant production itself. Eventually, high mountains serve as a geographical blockade and obstruct the species to relocate upwards due to the climatic condition atop the summit (Salick et al., 2009; Rana et al., 2017; Rana et al., 2019), because of summit trap phenomena (Pertoldi and Bach, 2007). This finally leads to the extinction of species due to no habitat for survival atop the mountain. Future distributions show the north-westward elevational shift of the species habitat and the dispersal corridor as well, which represents more credible conservation priority areas in the north-west higher elevational region of the HHM for *M. himalaica*. Geo-climatic variables are by all account not the only component to cause species habitat or population destruction because in present world anthropogenic factors are equally contributing to the cause. In this way, predicted expansion regions could be prioritized to protect the species from serious destruction of genetic diversity.

## Data Availability Statement

The haplotype sequence data have been deposited in GenBank with accessions: petL-*psb*E: MK792906–MK792916; *rps*16-*trn*K: MK792917–MK792925; *trn*S-*trn*G: MK792926–MK792935; *rps*16 *intron*: MK803108–MK803115; *G3pdh*: MK803090–MK803107. The matrix containing the geographic and climatic information used for species distribution modelling and the aligned sequence data matrix for both the markers were deposited in Dryad dataset (https://doi.org/10.5061/dryad.0zpc866t8).

## Author Contributions

HR and HS planned and designed the research. HR and SR carried out sampling and performed experiments. HR and DL analyzed data. HR and DL conceived the manuscript with the support of SR and HS.

## Conflict of Interest

The authors declare that the research was conducted in the absence of any commercial or financial relationships that could be construed as a potential conflict of interest.

## Supplementary Material

Supplementary Figure S1 Distributions of the *F*_CT_ values for the indicated number of groups (K) of *M. himalaica* populations based on cpDNA (solid line) and *G3pdh* (dotted line) sequences.

Supplementary Figure S2 Mismatch distribution of chloro/haplotypes of cpDNA/*G3pdh* in *M. himalaica*. The continuous lines with box represent observed distributions, whereas dotted lines with box represent simulated distributions under models of Demographic expansion (A and C) and spatial expansion (B and D) for cpDNA and *G3pdh* sequences, respectively (Rogers and Harpending, 1992).

Supplementary Figure S3 Historical demographic trends of the whole population sample of *M. himalaica* by Bayesian skyline plot (BSP) based on each marker and the combined (cpDNA and *G3pdh*) data. The x-axis in the plot represents time-scale before present, and the y-axis represents the estimated effective population size. Estimates of means are joined by a solid line, and the shaded range delineates the 95% HPD limits.

Supplementary Table S1 Details of population localities including population codes, voucher, geographic origins, coordinates (latitude and longitude), and altitudes of *M. himalaica*.

Supplementary Table S2 Pearson’s correlation (r) matrix performed among the 19 bioclimatic variables.

Supplementary Table S3 Result generated by Principal Components (PC) for the subset of explanatory variables, representing Eigenvalue and its percent.

Supplementary Table S4 Result of Model accuracy assessment for different General Circulation models (GCMs) under an ensemble distribution modelling using *Biomod2* in R-programming language.

Supplementary Table S5 cpDNA sequence polymorphisms detected in the combined region of *M. himalaica* individuals from 29 populations, identifying 29 chlorotypes (C1–C29).

Supplementary Table S6 Polymorphisms detected in the low-copy nuclear gene (*G3pdh*) region of *M. himalaica* individuals from 29 populations, identifying 18 haplotypes (H1–H18).

Supplementary Table S7 List of variables used as inputs to generate ensemble distribution models for Ecological Niche Modelling of *M. himalaica*.

Supplementary Method S1 Details on methods.
