# Supplementary figures and images for: Geological and Climatic Factors Affect the Population Genetic Connectivity in Mirabilis himalaica (Nyctaginaceae): Insight From Phylogeography and Dispersal Corridors in the Himalaya-Hengduan Biodiversity Hotspot
Source: Front Plant Sci. 2020 Jan 31;10:1721. doi: 10.3389/fpls.2019.01721 (PMC7006540; doi:10.3389/fpls.2019.01721)

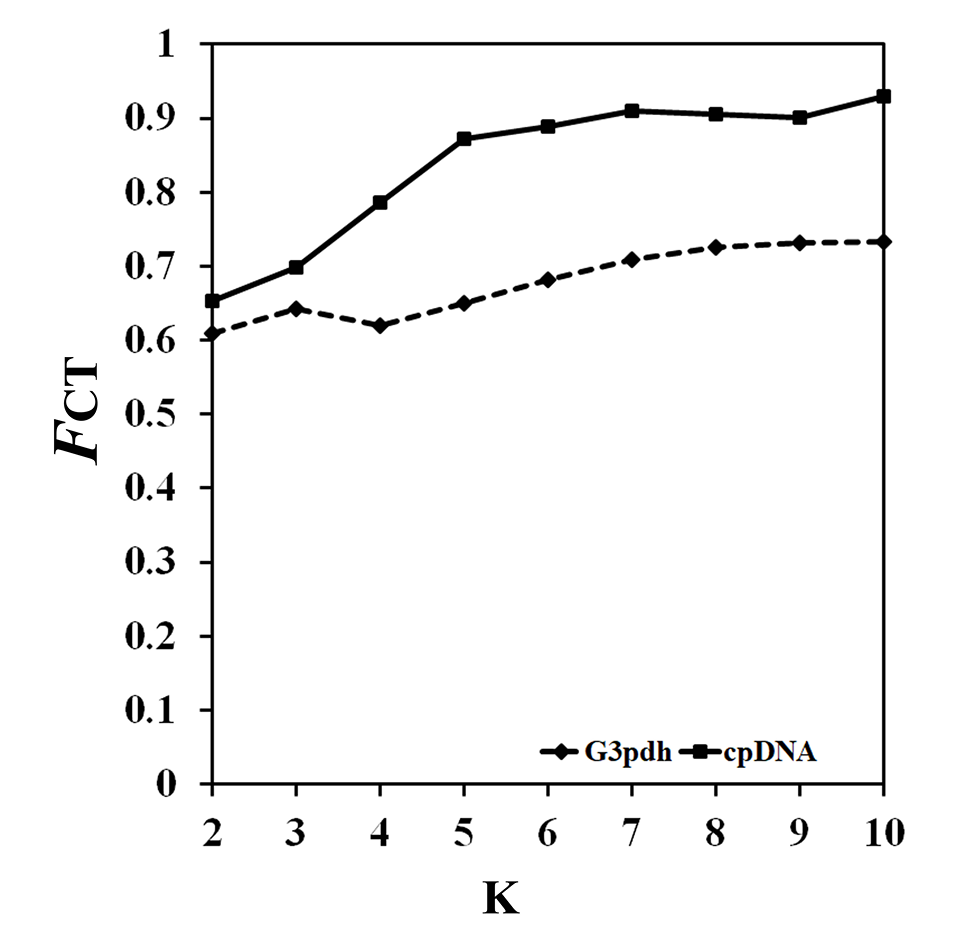

Supplement: Supplementary Figure S1 — Distributions of the F CT values for the indicated number of groups (K) of M. himalaica populations based on cpDNA (solid line) and G3pdh (dotted line) sequences. [file Image_1.jpeg]

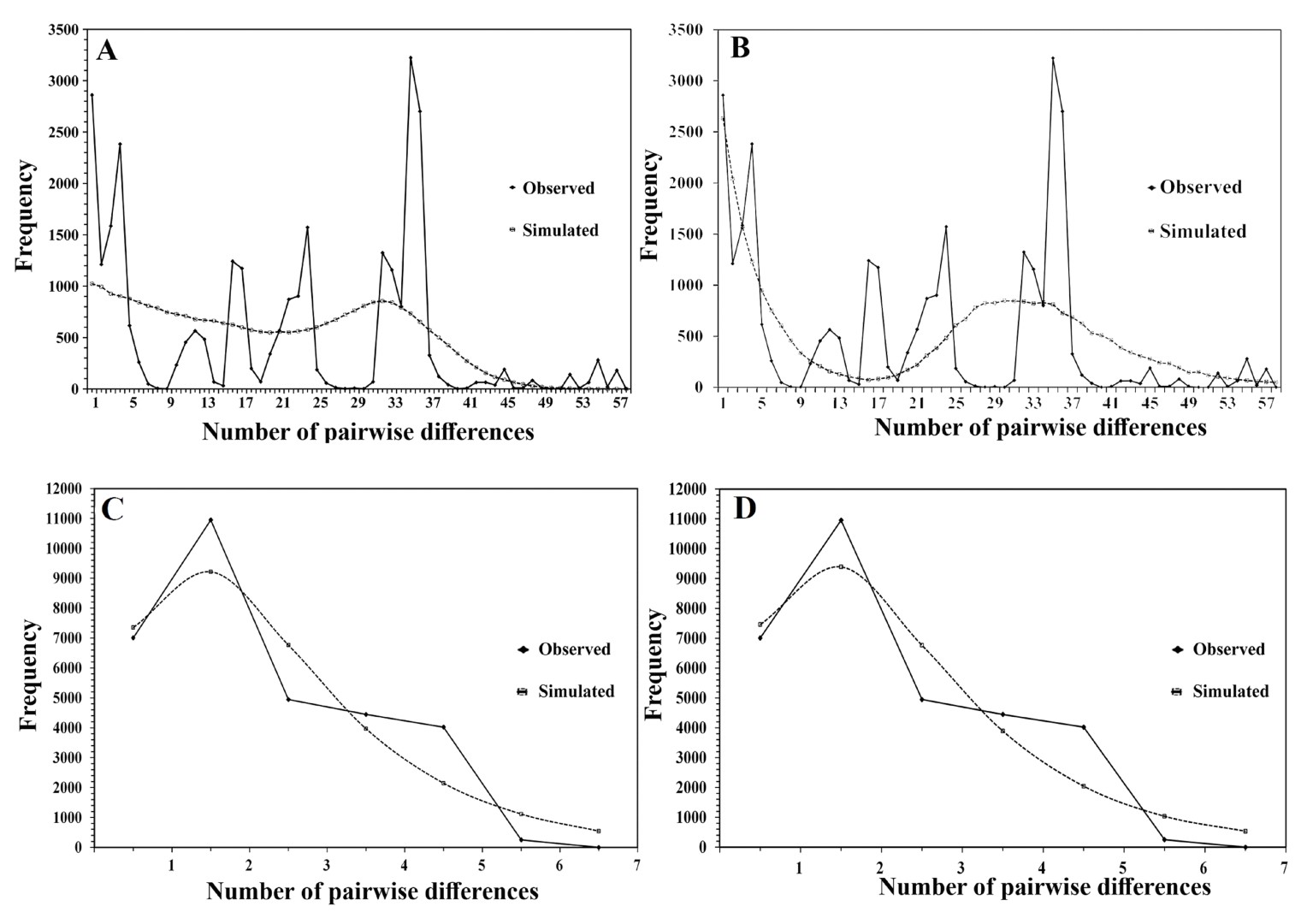

Supplement: Supplementary Figure S2 — Mismatch distribution of chloro/haplotypes of cpDNA/G3pdh in M. himalaica. The continuous lines with box represent observed distributions, whereas dotted lines with box represent simulated distributions under models of Demographic expansion (A and C) and spatial expansion (B and D) for cpDNA and G3pdh sequences, respectively (Rogers and Harpending, 1992). [file Image_2.jpeg]

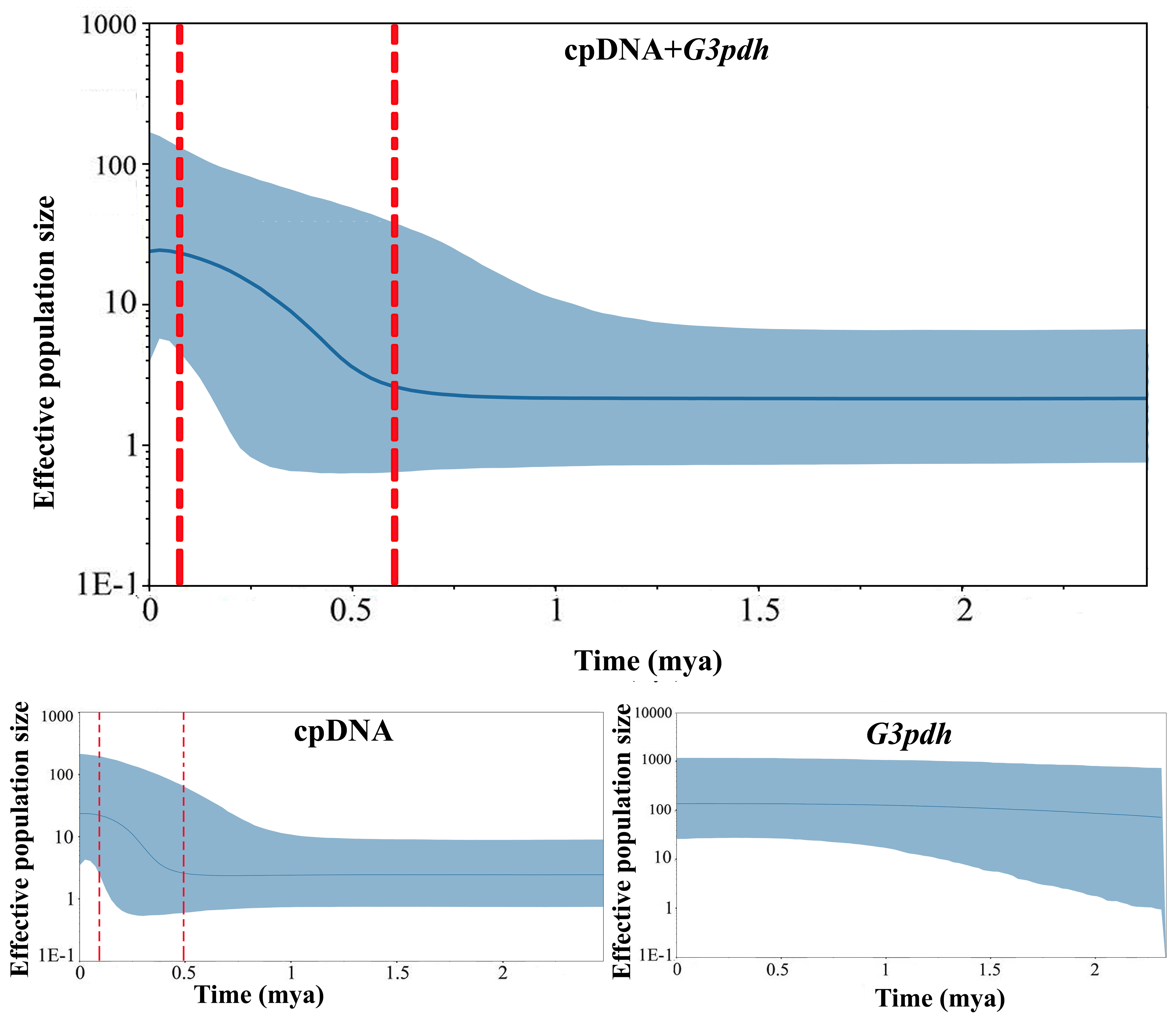

Supplement: Supplementary Figure S3 — Historical demographic trends of the whole population sample of M. himalaica by Bayesian skyline plot (BSP) based on each marker and the combined (cpDNA and G3pdh) data. The x-axis in the plot represents time-scale before present, and the y-axis represents the estimated effective population size. Estimates of means are joined by a solid line, and the shaded range delineates the 95% HPD limits. [file Image_3.jpeg]
