# Supplementary material for: Geological and Climatic Factors Affect the Population Genetic Connectivity in Mirabilis himalaica (Nyctaginaceae): Insight From Phylogeography and Dispersal Corridors in the Himalaya-Hengduan Biodiversity Hotspot
Source: Front Plant Sci. 2020 Jan 31;10:1721. doi: 10.3389/fpls.2019.01721 (PMC7006540; doi:10.3389/fpls.2019.01721)
Supplement: Supplementary Table S1 — Details of population localities including population codes, voucher, geographic origins, coordinates (latitude and longitude), and altitudes of M. himalaica. [file Table_1.doc]

**Supplementary Table S1**. Details of population localities including population codes, voucher, geographic origins, coordinates (latitude and longitude), and altitudes of *M. himalaica*.

| Population | Locality | Voucher | N | Latitude (N) | Longitude (E) | Altitude (m a.s.l.) |
| --- | --- | --- | --- | --- | --- | --- |
| **HM Group**  FY | Dali, YN, CN | Deng4536 |  | 25°38'07" | 100°19'33" | 1979 |
|  |  |  | 8 |  |  |  |
| GS | Derong, SC, CN | Deng4618 | 10 | 28°14'09.65" | 99°18'23.80" | 2005 |
| YY | Yong Zhi, Deqin, YN, CN | Deng4641 | 8 | 28°11'52.56" | 98°51'49.05" | 1903 |
| MY | Ming Yong, Deqin, YN, CN | Deng4647 | 10 | 28°28'39" | 98°49'06" | 2127 |
| CT | Qamdo, Mangkang, XZ, CN | Deng4657 | 8 | 29°37'21.96" | 98°21'10.90" | 2616 |
| DMT | Deng Ba, Mangkang, XZ, CN | Deng4672 | 6 | 29°32'21.76" | 98°14'34.28" | 3454 |
| MT | Mirandaville, Basu, XZ, CN | Deng4680 | 8 | 30°05'10.92" | 97°17'26.86" | 3054 |
| WS | Maerkang, SC, CN | Deng4845 | 6 | 31°50'59.49" | 101°45'28.03" | 2446 |
| JS | Jinchuan, SC, CN | Deng4856 | 6 | 31°29'36.57" | 102°4'2.2" | 2161 |
| XJS | Xiaojin, SC, CN | Deng4857 | 6 | 30°59'29.56" | 102°34'56.58" | 2609 |
| LS | ABA, Li, SC, CN | Deng4905 | 8 | 31°29'04.60" | 103°11'52.21" | 1732 |
| HS | Heishui, SC, CN | Deng4906 | 8 | 32°4'13.99" | 103°3'56.72" | 2267 |
| MS | Mao, SC, CN | Deng4907 | 10 | 31°53'55.43" | 103°26'31.92" | 1812 |
| KS | Kangding, SC, CN | Deng4911 | 6 | 30°04'14.50" | 102°07'54.52" | 1484 |
| YS | Yajiang, SC, CN | Deng4914 | 6 | 30°02'04.95" | 101°01'22.98" | 2579 |
| DS | Daocheng, SC, CN | Deng4929 | 8 | 28°49'20.50" | 100°29'05.78" | 3042 |
| XCS | Xiangcheng, SC, CN | Deng4932 | 7 | 29°07'10.06" | 99°54'35.27" | 2990 |
| DY | Near Deqin, YN, CN | Deng4629 | 10 | 28°28'44.25" | 98°55'09.40" | 3153 |
| **QTP Group** |  |  |  |  |  |  |
| QT | Qushui, Lasa, XZ, CN | Deng4757 | 6 | 29°15'30.44" | 90°31'16.19" | 3599 |
| WT | Wang Yao Shan, Lasa, XZ, CN | Deng4756 | 10 | 29°39'15.50" | 91°06'47.19" | 3628 |
| DT | Dazi, Lhasa, XZ, CN | Deng4771 | 6 | 29°40'14.43" | 91°20'12.17" | 3661 |
| **WHN Group** |  |  |  |  |  |  |
| CJ | Chandannath, JL, NP | SH-71 | 10 | 29°16'24" | 82°11'32" | 2346 |
| LJ | Lamra to Kalikot, JL, NP | SH-78 | 10 | 29°13'52" | 82°2'48" | 2273 |
| JM | Jomsom to Marpha, MT, NP | SH-65 | 10 | 28°45'38" | 83°41'40" | 2745 |
| MM | Muktinath to Jhong, MT, NP | SH-58 | 10 | 28°49'57" | 83°50'17" | 3405 |
| KM | Kagbeni to Tangbe, MT, NP | SH-59 | 10 | 28°50'45" | 83°47'28" | 2913 |
| LM | Lo-Manthang, MT, NP | SH-63 | 10  10 | 29°10'46" | 83°57'30" | 3798 |
| TM | Tanki Manang, MN, NP | SH-53 |  | 28°39'56.8" | 84°1'18.13" | 3528 |
| **WHI Group** |  |  |  |  |  |  |
| RI | Recongpeo, HP, IN | SH-04 | 10 | 31°33'27.15" | 78°16'43.74" | 2021 |

Abbreviations: CN = China; NP = Nepal; IN = India; HP = Himachal Pradesh; JL = Jumla; MT = Mustang; MN = Manang; SC = Sichuan Province; XZ = Xizang Autonomous Region; YN = Yunnan Province.
