# Supplementary material for: Geological and Climatic Factors Affect the Population Genetic Connectivity in Mirabilis himalaica (Nyctaginaceae): Insight From Phylogeography and Dispersal Corridors in the Himalaya-Hengduan Biodiversity Hotspot
Source: Front Plant Sci. 2020 Jan 31;10:1721. doi: 10.3389/fpls.2019.01721 (PMC7006540; doi:10.3389/fpls.2019.01721)
Supplement: Supplementary Table S2 — Pearson’s correlation (r) matrix performed among the 19 bioclimatic variables. [file Table_2.docx]

**Supplementary Table S2.** Pearson’s correlation (r) matrix performed among the 19 bioclimatic variables.

| ***Var**** | Bio1 | **Bio2** | **Bio3** | **Bio4** | **Bio5** | Bio6 | Bio7 | Bio8 | Bio9 | Bio10 | Bio11 | Bio12 | Bio13 | **Bio14** | **Bio15** | Bio16 | **Bio17** | **Bio18** | Bio19 |
| --- | --- | --- | --- | --- | --- | --- | --- | --- | --- | --- | --- | --- | --- | --- | --- | --- | --- | --- | --- |
| Bio1 | 1.00 |  |  |  |  |  |  |  |  |  |  |  |  |  |  |  |  |  |  |
| **Bio2** | -0.39 | 1.00 |  |  |  |  |  |  |  |  |  |  |  |  |  |  |  |  |  |
| **Bio3** | -0.09 | 0.31 | 1.00 |  |  |  |  |  |  |  |  |  |  |  |  |  |  |  |  |
| **Bio4** | -0.27 | 0.57 | -0.59 | 1.00 |  |  |  |  |  |  |  |  |  |  |  |  |  |  |  |
| **Bio5** | 0.92 | -0.15 | -0.29 | 0.09 | 1.00 |  |  |  |  |  |  |  |  |  |  |  |  |  |  |
| Bio6 | 0.91 | -0.68 | -0.01 | -0.58 | 0.72 | 1.00 |  |  |  |  |  |  |  |  |  |  |  |  |  |
| Bio7 | -0.32 | 0.81 | -0.30 | 0.94 | 0.05 | -0.66 | 1.00 |  |  |  |  |  |  |  |  |  |  |  |  |
| Bio8 | 0.90 | -0.13 | -0.11 | -0.03 | 0.91 | 0.71 | -0.05 | 1.00 |  |  |  |  |  |  |  |  |  |  |  |
| Bio9 | 0.90 | -0.63 | 0.03 | -0.59 | 0.71 | 0.98 | -0.64 | 0.67 | 1.00 |  |  |  |  |  |  |  |  |  |  |
| Bio10 | 0.97 | -0.26 | -0.25 | -0.03 | 0.98 | 0.80 | -0.09 | 0.93 | 0.78 | 1.00 |  |  |  |  |  |  |  |  |  |
| Bio11 | 0.98 | -0.49 | 0.05 | -0.48 | 0.82 | 0.97 | -0.51 | 0.83 | 0.95 | 0.89 | 1.00 |  |  |  |  |  |  |  |  |
| Bio12 | 0.72 | -0.44 | -0.31 | -0.16 | 0.63 | 0.66 | -0.26 | 0.65 | 0.64 | 0.71 | 0.69 | 1.00 |  |  |  |  |  |  |  |
| Bio13 | 0.67 | -0.32 | -0.23 | -0.13 | 0.60 | 0.58 | -0.19 | 0.65 | 0.59 | 0.66 | 0.64 | 0.95 | 1.00 |  |  |  |  |  |  |
| **Bio14** | 0.57 | -0.66 | -0.31 | -0.35 | 0.42 | 0.66 | -0.48 | 0.32 | 0.72 | 0.50 | 0.60 | 0.76 | 0.66 | 1.00 |  |  |  |  |  |
| **Bio15** | 0.00 | 0.66 | 0.15 | 0.44 | 0.16 | -0.28 | 0.58 | 0.28 | -0.29 | 0.12 | -0.09 | -0.01 | 0.22 | -0.42 | 1.00 |  |  |  |  |
| Bio16 | 0.71 | -0.29 | -0.19 | -0.13 | 0.63 | 0.60 | -0.18 | 0.70 | 0.58 | 0.70 | 0.68 | 0.96 | 0.99 | 0.62 | 0.24 | 1.00 |  |  |  |
| **Bio17** | 0.37 | -0.72 | -0.28 | -0.45 | 0.23 | 0.57 | -0.58 | 0.06 | 0.65 | 0.26 | 0.44 | 0.54 | 0.46 | 0.80 | -0.57 | 0.38 | 1.00 |  |  |
| **Bio18** | 0.67 | -0.18 | -0.05 | -0.13 | 0.59 | 0.54 | -0.15 | 0.73 | 0.49 | 0.67 | 0.65 | 0.89 | 0.90 | 0.44 | 0.34 | 0.95 | 0.16 | 1.00 |  |
| Bio19 | 0.24 | -0.65 | -0.31 | -0.36 | 0.13 | 0.44 | -0.49 | -0.09 | 0.54 | 0.14 | 0.29 | 0.40 | 0.34 | 0.78 | -0.58 | 0.24 | 0.97 | 0.00 | 1.00 |

Variables with bold letters are the subset of explanatory variables used for model building. (r > 0.8 were excluded due to high collinearity).

*See Supplementary Table S7 for the abbreviated form of 19 bioclimatic variables.
