# Supplementary material for: Geological and Climatic Factors Affect the Population Genetic Connectivity in Mirabilis himalaica (Nyctaginaceae): Insight From Phylogeography and Dispersal Corridors in the Himalaya-Hengduan Biodiversity Hotspot
Source: Front Plant Sci. 2020 Jan 31;10:1721. doi: 10.3389/fpls.2019.01721 (PMC7006540; doi:10.3389/fpls.2019.01721)
Supplement: Supplementary Table S3 — Result generated by Principal Components (PC) for the subset of explanatory variables, representing Eigenvalue and its percent. [file Table_3.doc]

**Supplementary Table S3.** Result generated by Principal Components (PC) for the subset of explanatory variables, representing Eigenvalue and its percent.

|  | PC1 | PC2 | PC3 | PC4 |
| --- | --- | --- | --- | --- |
| Eigen value | 618800.00 | 121000.00 | 6696.42 | 4517.80 |
| Proportion Explained | 0.82 | 0.16 | 0.01 | 0.01 |
