# Supplementary material for: Geological and Climatic Factors Affect the Population Genetic Connectivity in Mirabilis himalaica (Nyctaginaceae): Insight From Phylogeography and Dispersal Corridors in the Himalaya-Hengduan Biodiversity Hotspot
Source: Front Plant Sci. 2020 Jan 31;10:1721. doi: 10.3389/fpls.2019.01721 (PMC7006540; doi:10.3389/fpls.2019.01721)
Supplement: Supplementary Table S4 — Result of Model accuracy assessment for different General Circulation models (GCMs) under an ensemble distribution modelling using Biomod2 in R-programming language. [file Table_4.doc]

**Supplementary Table S4**. Result of Model accuracy assessment for different Global climate models (GCMs) under an ensemble distribution modelling using *Biomod2* in R-programming language.

| Bioclimatic Model | LIG: ca. 120–140 Ka | | | LGM: ca. 22 Ka | | | | | | Present (1990 - 2000) | | | Future (2070; RCP 4.5) | | | | | |
| --- | --- | --- | --- | --- | --- | --- | --- | --- | --- | --- | --- | --- | --- | --- | --- | --- | --- | --- |
|  |  |  |  | CCSM4 | | | MIROC-ESM | | |  |  |  | CCSM4 | | | MIROC-ESM | | |
|  | KAPPA | TSS | AUC | KAPPA | TSS | AUC | KAPPA | TSS | AUC | KAPPA | TSS | AUC | KAPPA | TSS | AUC | KAPPA | TSS | AUC |
| GLM | 0.83 | 0.97 | 0.99 | 0.90 | 0.99 | 1.00 | 0.87 | 0.98 | 0.99 | 0.90 | 0.99 | 1.00 | 0.90 | 0.99 | 1.00 | 0.90 | 0.99 | 1.00 |
| GBM | 0.90 | 0.98 | 1.00 | 0.90 | 0.99 | 1.00 | 0.92 | 0.99 | 1.00 | 0.90 | 0.99 | 1.00 | 0.90 | 0.99 | 1.00 | 0.90 | 0.99 | 1.00 |
| GAM | 1.00 | 1.00 | 1.00 | 1.00 | 1.00 | 1.00 | 1.00 | 1.00 | 1.00 | 1.00 | 1.00 | 1.00 | 1.00 | 1.00 | 1.00 | 1.00 | 1.00 | 1.00 |
| CTA | 0.75 | 0.91 | 0.96 | 0.73 | 0.90 | 0.95 | 0.87 | 0.97 | 0.99 | 0.73 | 0.90 | 0.95 | 0.73 | 0.90 | 0.95 | 0.73 | 0.90 | 0.95 |
| ANN | 0.79 | 0.93 | 0.96 | 0.79 | 0.90 | 0.97 | 0.89 | 0.95 | 0.98 | 0.91 | 0.97 | 0.99 | 0.77 | 0.90 | 0.95 | 0.78 | 0.89 | 0.95 |
| SRE | 0.78 | 0.80 | 0.90 | 0.79 | 0.79 | 0.90 | 0.80 | 0.76 | 0.88 | 0.79 | 0.79 | 0.90 | 0.79 | 0.79 | 0.90 | 0.79 | 0.79 | 0.90 |
| FDA | 0.78 | 0.89 | 0.98 | 0.86 | 0.94 | 0.99 | 0.87 | 0.95 | 0.99 | 0.86 | 0.94 | 0.99 | 0.86 | 0.94 | 0.99 | 0.86 | 0.94 | 0.99 |
| MARS | 0.86 | 0.97 | 0.99 | 0.89 | 0.98 | 1.00 | 0.87 | 0.97 | 0.99 | 0.89 | 0.98 | 1.00 | 0.89 | 0.98 | 1.00 | 0.89 | 0.98 | 1.00 |
| RF | 0.99 | 1.00 | 1.00 | 0.98 | 1.00 | 1.00 | 0.98 | 1.00 | 1.00 | 0.98 | 1.00 | 1.00 | 0.98 | 1.00 | 1.00 | 0.98 | 1.00 | 1.00 |
| MAXENT | 0.79 | 0.77 | 0.83 | 0.78 | 0.77 | 0.83 | 0.89 | 0.82 | 0.91 | 0.78 | 0.77 | 0.83 | 0.78 | 0.77 | 0.83 | 0.78 | 0.77 | 0.83 |

Refer to text for the description of the two different GCMs and 10 different bioclimatic models.

AUC *=* Area Under the Curve; TSS *=* True Skill Statistics; Kappa = *Cohen's kappa*; LIG = Last Inter-glacial; LGM *=* Last Glacial Maximum; *RCP 4.5* Representative Concentration Pathway.
