# Supplementary material for: Geological and Climatic Factors Affect the Population Genetic Connectivity in Mirabilis himalaica (Nyctaginaceae): Insight From Phylogeography and Dispersal Corridors in the Himalaya-Hengduan Biodiversity Hotspot
Source: Front Plant Sci. 2020 Jan 31;10:1721. doi: 10.3389/fpls.2019.01721 (PMC7006540; doi:10.3389/fpls.2019.01721)
Supplement: Supplementary Table S6 — Polymorphisms detected in the low-copy nuclear gene (G3pdh) region of M. himalaica individuals from 29 populations, identifying 18 haplotypes (H1–H18). [file Table_6.doc]

**Supplementary Table S6.** Polymorphisms detected in the low-copy nuclear gene (*G3pdh*) region of *M. himalaica* individuals from 29 populations, identifying 18 haplotypes (H1–H18).

| Haplotype | *G3pdh* | | | | | | | | | | | | | | | | | | | |
| --- | --- | --- | --- | --- | --- | --- | --- | --- | --- | --- | --- | --- | --- | --- | --- | --- | --- | --- | --- | --- |
|  | 3 | 50 | 146 | 168 | 200 | 208 | 251 | 255 | 278 | 374 | 376 | 400 | 408 | 440 | 554 | 672 | 680 | 722 | 843 | 876 |
| H1 | A | G | G | C | T | C | G | C | A | G | C | A | G | T | C | T | T | C | C | C |
| H2 | . | . | . | . | . | . | . | . | . | . | . | . | . | C | . | . | . | . | . | . |
| H3 | . | . | . | . | . | . | . | . | . | . | . | G | . | C | . | . | . | . | . | . |
| H4 | . | . | . | . | . | . | . | T | . | . | . | . | . | . | . | . | . | . | . | . |
| H5 | . | . | . | . | . | . | . | T | . | . | A | . | . | . | . | . | . | . | . | . |
| H6 | . | . | . | T | . | . | . | . | . | . | . | . | . | . | . | . | . | . | . | . |
| H7 | . | . | . | . | . | . | . | . | . | . | . | . | . | . | . | . | G | . | T | . |
| H8 | . | . | . | . | . | . | . | . | . | . | . | . | A | . | . | . | . | . | . | . |
| H9 | . | . | . | . | . | . | . | . | . | . | . | . | . | . | . | C | . | . | . | . |
| H10 | . | . | . | . | . | . | . | . | . | . | . | . | . | . | . | . | . | . | T | . |
| H11 | . | . | A | . | . | . | . | . | . | A | . | . | . | . | . | . | . | . | . | . |
| H12 | G | . | . | . | . | . | . | . | . | A | . | . | . | . | . | . | . | . | . | . |
| H13 | . | . | . | . | . | . | . | . | . | . | . | . | . | . | T | . | . | . | . | . |
| H14 | . | . | . | . | . | . | C | . | . | . | . | . | . | . | . | . | . | . | . | . |
| H15 | . | . | . | . | C | . | . | . | . | . | . | . | . | . | . | . | . | T | . | G |
| H16 | . | . | . | . | . | . | . | . | G | . | . | . | . | . | . | . | . | . | . | . |
| H17 | . | T | . | . | . | T | . | . | . | . | . | . | . | . | . | . | . | T | . | . |
| H18 | . | . | . | . | . | . | . | . | . | . | . | . | . | . | . | . | . | T | . | . |

All sequences are compared to reference haplotype H1. ‘.’ indicates characterstates identical as in H1.
