# Supplementary material for: Geological and Climatic Factors Affect the Population Genetic Connectivity in Mirabilis himalaica (Nyctaginaceae): Insight From Phylogeography and Dispersal Corridors in the Himalaya-Hengduan Biodiversity Hotspot
Source: Front Plant Sci. 2020 Jan 31;10:1721. doi: 10.3389/fpls.2019.01721 (PMC7006540; doi:10.3389/fpls.2019.01721)
Supplement: Supplementary Table S7 — List of variables used as inputs to generate ensemble distribution models for Ecological Niche Modelling of M. himalaica. [file Table_7.doc]

**Supplementary Table S7**. List of variables used as inputs to generate ensemble distribution models for Ecological Niche Modelling of *M. himalaica*.

| Abbreviation | Name of bioclimatic variable |
| --- | --- |
| Bio1 | Annual Mean Temperature |
| **Bio2** | Mean Diurnal Range (Mean of monthly (max temp - min temp)) |
| **Bio3** | Isothermality (Bio2/Bio7) (* 100) |
| **Bio4** | Temperature Seasonality (standard deviation *100) |
| **Bio5** | Max Temperature of Warmest Month |
| Bio6 | Min Temperature of Coldest Month |
| Bio7 | Temperature Annual Range (Bio5-Bio6) |
| Bio8 | Mean Temperature of Wettest Quarter |
| Bio9 | Mean Temperature of Driest Quarter |
| Bio10 | Mean Temperature of Warmest Quarter |
| Bio11 | Mean Temperature of Coldest Quarter |
| Bio12 | Annual Precipitation |
| Bio13 | Precipitation of Wettest Month |
| **Bio14** | Precipitation of Driest Month |
| **Bio15** | Precipitation Seasonality (Coefficient of Variation) |
| Bio16 | Precipitation of Wettest Quarter |
| **Bio17** | Precipitation of Driest Quarter |
| **Bio18** | Precipitation of Warmest Quarter |
| Bio19 | Precipitation of Coldest Quarter |

Bold letter variables are used as a subset of explanatory variables for model building.

Source: *www.worldclim.org/bioclim;* Hijmans et al.*,* 2005

Temperature unit: °C (degree Celcius); Precipitation unit: millimeter (mm)
