# Supplementary material for: Geological and Climatic Factors Affect the Population Genetic Connectivity in Mirabilis himalaica (Nyctaginaceae): Insight From Phylogeography and Dispersal Corridors in the Himalaya-Hengduan Biodiversity Hotspot
Source: Front Plant Sci. 2020 Jan 31;10:1721. doi: 10.3389/fpls.2019.01721 (PMC7006540; doi:10.3389/fpls.2019.01721)
Supplement: Supplementary Method S1 — Details on methods. [file DataSheet_1.docx]

**Supplementary Method S1** Details on methods.

**DNA extraction, PCR amplification, and sequencing**

Total genomic DNA was extracted from c. 30 mg silica dried leaves using a DNA Plant Kit (Tiangen Biotechnology Co. Ltd., Beijing, China) following the manufacturer's protocol. After preliminary screening of 15 cpDNA regions (*atp*I-*atp*H, *pet*L-*psb*E, *psb*J-*pet*A, *rpl*32-*trn*L, *rps*12-*rpl*20, *rps*16 intron, *rps*16-*trn*K, *trn*L-*trn*F, *trn*S-*trn*fM, *trn*S-*trn*G, *trn*Q-*rps*16, *ndh*C-*trn*V, *ndh*F, *rbcl*, *mat*K), the internal transcribed spacer (ITS) and external transcribed spacer (ETS) regions of the nuclear DNA, and seven low copy nuclear genes (*G3pdh, ncpGS, pepC, Cam, Chi, Chs, TTP*) using primers published elsewhere (White et al., 1990; Taberlet et al., 1991; Wolf, 1995; Strand et al., 1997; Oxelman et al., 1997; Baldwin and Markos, 1998; Emshwiller and Doyle, 1999; Whittall et al., 2006; Shaw et al., 2007; Dong et al., 2012). Finally, we amplified four cpDNA regions i.e. *pet*L-*psb*E*, rps*16-*trn*K, *rps*16 *intron,* and *trn*S-*trn*G, and a low copy nuclear gene i.e. *G3pdh* for each individual.

PCRs were conducted in a 30 µL containing 2 µL DNA template (10–50 ng/µL), 15 µL 2x Taq Plus Master Mix with dye (Tiangen Biotech.),1 µL 10 µM of each primer. All PCR reactions for cpDNA were performed as followed: initial denaturation of 5 min at 80°C; followed by 32 cycles of 1 min at 95°C, 1 min at 50°C, an increase of 15°C in 1°C increments of 3 sec each to, and at 65°C for 4 min; with a final extension at 65°C for 8 min. The thermal condition for *G3pdh* with an initial denaturation of 2 min at 95°C; followed by 35 cycles of 1 min at 95°C, 90 sec at 50°C, and for 2 min at 72°C; with a final extension for 9 min at 72°C for *G3pdh* gene. PCR products were purified and sequenced by Tsingke Biological Technology Co. (Beijing, China).

**Genetic structure analyses**

Spatial analysis of molecular variance (Samova v2.0; Dupanloup et al., 2002), was implemented to define the number of groups of populations (*K*) vary from 2 to 10, that are geographically homogeneous and maximally differentiated from each other; the number of initial conditions was set to 100, and the pairwise difference was chosen as molecular distance. An *F*_CT_ index of genetic differentiation (Wright, 1978) was then computed for the initial *K* values, followed by an iterative simulated annealing process to obtain the optimal configuration of groups and final *F*_CT_.

**Phylogenetic analyses and divergence dating**

Phylogenetic relationships among chlorotypes of cpDNA, along with three closest species of *Mirabilis* L. (*M. jalapa*, EF079612; *M. multiflora*, EF079603; *M. albida*, EF079602/KR014118) as outgroups were constructed by Bayesian inference (BI) in MrBayes v3.2 (Ronquist et al., 2012) and Beast v1.8.4 (Drummond and Rambaut, 2007; Drummond et al., 2012) under the GTR+I nucleotide substitution model selected by Akaike information criterion (AIC) in jModelTest v2.1.6 (Guindon and Gascuel, 2003; Posada and Buckley, 2004). Markov chain Monte Carlo (MCMC) algorithm was run for 20,000,000 generations starting from random trees and sampling every 1,000 generations. The first 20% of the trees were discarded as burn-in followed by reconstruction of the majority consensus tree internodes with posterior probabilities as per Huelsenbeck and Ronquist (2001).

Beast v1.8.4 (Drummond and Rambaut, 2007; Drummond et al., 2012) was employed to estimate the temporal intraspecific divergence times (crown ages) of chlorotypes/haplotypes. We assumed a strict clock (*P =* 0.85; i.e. *P* > 0.05), based on a likelihood-ratio test (Felsenstein, 1988) in Paup* 4.0b10 (Swofford, 2002) and constant population size for the coalescent tree prior to the distribution of divergence times. We used 13.13 Ma (Node A, Figure 2; 95% highest posterior density [HPD]: 6.91–20.62 Ma) as first calibration point because *M. albida* (outgroup) diverged first at this age; and second calibration point c. 5.22 Ma (Node B, Figure 2; 95% HPD: 2.53–8.18 Ma) at which *M. himalaica* diverged from its North American counterparts according to Wang et al. (2019). Three independent runs of 20,000,000 generations were carried out, with sampling every 1000 generations, and the first 20% of the trees were discarded as burn-in. TreeAnnotator v1.8.4 (Drummond and Rambaut, 2007; Drummond et al., 2012) was used to obtain the best tree merging and FigTree v1.4.0 (Rambaut, 2012) was employed to view the resulting tree.

**Ensemble Species Distribution Modelling**

**Species location data**

The location data of *Mirabilis himalaica* was compiled from herbarium specimen housed in the National Herbarium and Plant Laboratories (KATH), Kathmandu, Nepal and Kunming Institute of Botany, CAS (KUN), Kunming, China, as well as our field study (Supplementary Table S1). In addition, online databases of Chinese National Herbarium (PE; http://pe.ibcas.ac.cn/en/), Chinese Virtual Herbarium (CVH; http://www.cvh.ac.cn/), Global Biodiversity Information Facility (GBIF; https://www.gbif.org/), the Royal Botanical Garden at Edinburgh (RBGE; http://data.rbge.org.uk/search/herbarium/), United Kingdom and the Herbarium at the University of Tokyo (TI; http://umdb.um.u-tokyo.ac.jp/DShokubu/), Japan were accessed for databasing the species location. All the unknown coordinates from herbarium were georeferenced carefully based on the locality to obtain a maximum number of occurrence points. The duplicates occurrence points were deleted and checked in different spatial grids to prevent from geo-coding errors and spatial autocorrelation (Rana et al., 2019). Finally, 72 unique occurrence points at 4-min grid (Rana et al., 2017) and 1000 random background points within an area of 50 km radius from the point of occurrence (Barbet-Massin et al., 2012) throughout the Himalaya-Hengduan Mountain (HHM) were used for the model building.

**Bioclimatic data**

The ensemble distribution of *M. himalaica* was modeled for the current (1990–2000) and then projected to paleoclimatic conditions using a LIG (~ 120–140 Ka; Otto-Bliesner et al., 2006) and two LGM (~ 22 Ka) General Circulation Models (GCMs) provided by Paleoclimate Modelling Intercomparisons Project Phase 5 (CMIP5; Braconnot et al., 2007). Likewise, the present distribution model was also projected to the future (2070) under the two GCMs model provided by CMIP5. There are four representative concentration pathways (RCP; van Vuuren et al., 2011), ranging from RCP 2.6 (aggressive mitigation/lowest emissions) to RCP 8.5 (highest emissions scenario) within each GCM of future scenario. Among the four RCPs, the selection of RCP 4.5 as a future scenario is based on its stabilization without overshoot beyond 2100 (Moss et al., 2010; Luo et al., 2018). The two models were CCSM4 and MIROC-ESM (Supplementary Table S4). All these 19 bioclimatic layers of the WorldClim databases (Hijmans et al., 2005; www.worldclim.org) (Supplementary Table S7) were used to obtain predictive explanatory variables of *M. himalaica* distribution with a spatial resolution of 2.5 arc-min.

**Selection of predictor variables**

The predictive explanatory variables were selected from the Worldclim datasets (Hijmans et al., 2005) of 19 bioclimatic layers (Bio1–Bio19) at 2.5 arc-min resolution based on Variance Inflation Factor (VIF; Fox and Weisberg, 2011), Pearson correlation (r), followed by confirmatory PCA-test. The high collinearity among the bioclimatic variables makes difficult to perceive the effects of predictors in the ensemble modelling approach (Ranjitkar et al., 2014). Therefore, we used the VIF in combination with the Pearson correlation matrix to test multi-collinearity and remove highly correlated redundancy variables. The bioclimatic variables were selected based on iterative calculations of variance-inflation factors (VIF; Fox and Weisberg, 2011; Ranjitkar et al., 2014), where VIF values > 10 were eliminated (Table 2) to list a subset of the least correlated predictor variables using ‘car’ package implemented in R-programming language (R 3.4.1; R Development Core Team, 2016). The variables were also checked for Pearson correlation and the final subset of predictors has correlation values r < 0.8 (Supplementary Table S2). The confirmation of the predictive final subset of the variable was done by Principal component analysis (PCA) analysis in R-Programming (Supplementary Table S3). Finally, a total of eight bioclimatic variables i.e. Bio 2, Bio 3, Bio 4, Bio 5, Bio 14, Bio 15, Bio 17, and Bio 18 were used as a subset of explanatory variables (Supplementary Table S7).

**Species distribution models**

An ensemble of Species Distribution Modellings (SDMs; Guisan and Zimmermann, 2000) was carried out in “*Biomod 2*” (Thuiller et al., 2014) package implemented in R v3.4.1 software (R Development Core Team, 2016) for the present, past (LIG and LGM), and future. The assessment of ensemble species distribution on niche-based modelling techniques allows running different modelling techniques as bioclimatic envelopes, regression, classification methods, and machine learning methods (Thuiller et al., 2014) (Supplementary Table S4). This assessment uses default parameters for all the ten simulated distribution models; ANN (Artificial Neural Networks; Ripley, 1996), CTA (Classification Tree Analysis; Breiman, 2001; 2017), FDA (Flexible Discriminant Analysis; Hastie et al., 1994), GAM (Generalized Additive Models; Hastie and Tibshirani, 1990), GBM (Generalized Boosting Models; Ridgeway, 2006), GLM (Generalized Linear Models; Peter and John, 1989), MARS (Multiple Adaptive Regression Splines; Friedman, 1991), RF (Breiman and Cutlers Random Forest for classification and regression; Breiman, 2001), SRE (Surface Range Envelops; Busby, 1991), and MaxEnt (Maximum Entropy Models; Phillips et al., 2006). The regularization parameter for MaxEnt was changed to 5000 to avoid overfitting of the model.

The models were calibrated and evaluated on 25% of the data using TSS (True Skill Statistics), KAPPA and AUC (Area Under Curve-Receiver Operating characteristics) statistics. The sampling procedure was replicated for 6 times and the ensembles were generated using the model that had TSS ≥ 0.70, KAPPA ≥ 0.70, AUC ≥ 0.80. The consensus model was then projected onto the past (LIG, LGM) and future (2070) climatic scenarios under different GCMs. The contribution of each model to the final ensemble model was proportional to their goodness-of-fit statistics. Thus, obtained consensus ensemble model was converted to a binary model i.e. unsuitable/absence (0) and suitable/presence (1) (Figure 3) applying threshold that allows a maximum of 10%, 30%, 50%, and 70% probability of suitable habitat (Forester et al., 2013). Finally, we used a 70% probability of the suitable habitat that suits the present distribution of the focal species. The use of a high threshold is due to the inclusion of the MaxEnt model in the approach that generally overfits the models. We also reclassified changes in LIG compared to LGM, LGM and future conditions compared to current suitability into stable, retracted and expanded areas (Figure 4) using Raster Calculator as a function of spatial analysis.

**References**

Baldwin, B. G., and Markos, S. (1998) Phylogenetic utility of the external transcribed spacer (ETS) of 18S–26S rDNA: congruence of ETS and ITS trees of *Calycadenia* (Compositae). *Mol. Phylogenet. Evol.* 10, 449–463. doi: 10.1006/mpev.1998.0545

Barbet-Massin, M., Jiguet, F., Albert, C. H., and Thuiller, W. (2012). Selecting pseudo-absences for species distribution models: how, where and how many? *Methods in Ecol. Evol.* 3, 327–338. doi: 10.1111/j.2041-210X.2011.00172.x

Braconnot, P., Otto-Bliesner, B., Harrison, S., Joussaume, S., Peterchmitt, J. Y., Abe-Ouchi, A., Crucifix, M., Driesschaert, E., Fichefet, T., Hewitt, C. D., Kageyama, M., Kitoh. A., Laine, A., Loutre, M. F., Marti, O., Merkel, U., Ramstein, G., Valdes, P., Weber, S. L., Yu, Y., and Zhao, Y. (2007). Results of PMIP2 coupled simulations of the Mid-Holocene and Last Glacial Maximum-Part 1: experiments and large-scale features. *Clim. Past* 3 (2), 261–277.

Breiman, L. (2001). Random forests. *Mach. Learn.* 45, 5–32. doi: 10.1023/A:1010933404324

Breiman, L. (2017). *Manual on setting up, using, and understanding random forests V3.1*. [WWW document] URL http://www.stat.berkeley.edu/~breiman. [Accessed on 20 October 2018].

Busby, J. R. (1991). “Bioclim-a bioclimatic analysis and prediction system,” in *Nature Conservation: Cost Effective Biological Surveys and Data Analysis*, ed. C. R., Margules and M. P., Austin (Melbourne, CSIRO), 64–68.

Dong, W. P., Liu, J., Yu, J., Wang, L., and Zhou, S. L. (2012). Highly Variable Chloroplast Markers for Evaluating Plant Phylogeny at Low Taxonomic Levels and for DNA Barcoding. *PLoS ONE* 7 (4), e35071. doi: 10.1371/journal.pone.0035071

Drummond, A. J., and Rambaut, A. (2007). BEAST: Bayesian evolutionary analysis by sampling trees. *BMC Evol. Biol.* 7, 214. doi: 10.1186/1471-2148-7-214

Drummond, A. J., Suchard, M. A., Xie, D., and Rambaut, A. (2012). Bayesian phylogenetics with BEAUti and the BEAST 1.7. *Mol. Biol. Evol.* 29 (8), 1969–1973. doi: 10.1093/molbev/mss075

Dupanloup, I., Schneider, S., and Excoffier, L. (2002). A simulated annealing approach to define the genetic structure of populations. *Mol. Ecol.* 11, 2571– 2581. doi: 10.1046/j.1365-294X.2002.01650.x

Emshwiller, E., and Doyle, J. J. (1999). Chloroplast-expressed glutamine synthetase (ncpGS): potential utility for phylogenetic studies with an example from *Oxalis* (Oxalidaceae). *Mol. Phylogenet. Evol.* 12, 310–319. doi: 10.1006/mpev.1999.0613

Felsenstein, J. (1988). Phylogenies from molecular sequences: inference and reliability. *Annu. Rev. Genet.* 22, 521–565. doi: 10.1146/annurev.ge.22.120188.002513

Forester, B. R., DeChaine, E. G., and Bunn, A. G. (2013). Integrating ensemble species distribution modelling and statistical phylogeography to inform projections of climate change impacts on species distributions. *Divers. Distrib.* 19, 1480–1495. doi: 10.1111/ddi.12098

Fox, J., and Weisberg, S. (2011). An R Companion to Applied Regression, second ed. Thousand Oaks, CA: Sage.

Friedman, J. H. (1991). Multivariate adaptive regression splines. *Ann. Stat.* 19 (1), 1–67.

Guindon, S., and Gascuel, O. (2003). A simple, fast and accurate algorithm to estimate large phylogenies by maximum likelihood. *Syst. Biol.* 52 (5), 696–704. doi: 10.1080/10635150390235520

Guisan, A., and Zimmermann, N. E. (2000). Predictive habitat distribution models in ecology. *Ecol. Model.* 135, 147–186. doi: 10.1016/S0304-3800(00)00354-9

Hastie, T. J., and Tibshirani, R. J. (1990). *Generalized Additive Models*. New York: Chapman & Hall.

Hastie, T., Tibshirani, R., and Buja, A. (1994). Flexible discriminant analysis by optimal scoring. *J. Am. Stat. Assoc.* 89 (428), 255–1270. doi: 10.1080/01621459.1994.10476866

Hijmans, R. J., Cameron, S. E., Parra, J. L., Jones, P. G., and Jarvis, A. (2005). Very high-resolution interpolated climate surfaces for global land areas. *Int. J. Climatol.* 25, 1965–1978. doi: 10.1002/joc.1276

Huelsenbeck, J. P., and Ronquist, F. (2001). MRBAYES: bayesian inference of phylogenetic trees. *Bioinformatics* 17, 754–755. doi: 10.1093/bioinformatics/17. 8.754

Luo, D., Xu, B., Rana, S. K., Li, Z. M., and Sun, H. (2018). Phylogeography of rare fern *Polystichum glaciale* endemic to the subnival zone of the Sino-Himalaya. *Plant Syst. Evol.* 304, 485–499. doi: 10.1007/s00606-018-1495-2

Moss, R. H., Edmonds, J. A., Hibbard, K. A., Manning, M. R., Rose, S. K., van Vuuren, D. P., Carter, T. R., Emori, S., Kainuma, M., Kram, T., Meehl, G. A., Mitchell, J. F. B., Nakicenovic, N., Riahi, K., Smith, S. J., Stouffer, R. J., Thomson, A. M., Weyant, J. P., and Wilbanks, T. J. (2010). The next generation of scenarios for climate change research and assessment. *Nature* 463, 747–756. doi: 10.1038/nature08823

Oxelman, B., Liden, M., and Berglund, D. (1997). Chloroplast *rps*16 intron phylogeny of the tribe Sileneae (Caryophyllaceae). *Plant Syst. Evol.* 206, 393–410. doi: 10.1007/BF00987959

Peter, M. C., and John, N. (1989). *Generalized Linear Models*. Second ed. Boca Raton: Chapman and Hall/CRC.

Phillips, S. J., Anderson, R. P., and Schapire, R. E. (2006). Maximum entropy modelling of species geographic distributions. *Ecol. Model.* 190, 231–259. doi: 10.1016/j.ecolmodel.2005.03.026

Posada, D., and Buckley, T. (2004). Model selection and model averaging in phylogenetics: advantages of Akaike information criterion and Bayesian approaches over likelihood ratio tests. *Syst. Biol.* 53, 793–808. doi: 10.1093/bioinformatics/14.9.817

R Development Core Team. (2016). R v3.4.1: A language and environment for statistical computing. Vienna, Austria: R Foundation for Statistical Computing.

Rambaut, A. (2012). *FigTree, tree figure drawing tool v.1.4.0.* Institute of Evolutionary Biology, University of Edinburgh. [WWW document] URL http://tree.bio.ed.ac.uk/software/figtree/ [accessed 20 June 2018]

Rana, S. K., Luo, D., Rana, H. K., O’Neill, A. R., and Sun, H. (2019). Geoclimatic factors influence the population genetic connectivity of *Incarvillea arguta* (Bignoniaceae) in the Himalaya–Hengduan Mountains biodiversity hotspot. *J. Syst. Evol.* (Published online) doi: 10.1111/jse.12521

Rana, S. K., Rana, H. K., Ghimire, S. K., Shrestha, K. K., and Ranjitkar, S. (2017). Predicting the impact of climate change on the distribution of two threatened Himalayan medicinal plants of Liliaceae in Nepal. *J. Mt. Sci.* 14, 558–570. doi: 10.1007/s11629-015-3822-1

Ranjitkar, S., Xu, J., Shrestha, K. K., and Kindt, R. (2014). Ensemble forecast of climate suitability for the trans-Himalayan Nyctaginaceae species. *Ecol. Model.* 282, 18–24. doi: 10.1016/j.ecolmodel.2014.03.003

Ridgeway, G. (2006). GBM: generalized boosted regression models. Documentation on the R package ‘gbm’ (Version 1.5–7).

Ripley, B. D. (1996) *Pattern Recognition and Neural Networks.* Cambridge: Cambridge University Press.

Ronquist, F., Teslenko, M., Mark, P. V. D., Ayres, D., Darling, A., Höhna, S., Larget, B., Liu, L., Suchard, M. A., and Huelsenbeck, J. P. (2012). MrBayes 3.2: efficient Bayesian phylogenetic inference and model choice across a large model space. *Syst. Biol.* 61 (3), 539–542. doi: 10.1093/sysbio/sys029

Shaw, J., Lickey, E. B., Schilling, E. E., and Small, R. L. (2007). Comparison of whole chloroplast genome sequences to choose noncoding regions for phylogenetic studies in angiosperms: the tortoise and the hare III. *Am. J Bot.* 94, 275–288. doi: 10.3732/ajb.94.3.275

Strand, A. E., LeebensMack, J., and Milligan, B. G. (1997). Nuclear DNA-based markers for plant evolutionary biology. *Mol. Ecol.* 6, 113–118. doi: 10.1046/j.1365/294X.1997.00153.x

Swofford, D. L. (2002). *Paup*: Phylogenetic analysis using parsimony (and other methods)* v. 4.0b10. Sunderland, Massachusetts, USA: Sinauer Associates.

Taberlet, P., Gielly, L., Pautou, G., and Bouvet, J. (1991). Universal primers for amplification of three non-coding regions of chloroplast DNA. *Plant Mol. Biol.* 17, 1105–1109. doi: 10.1007/bf00037152

Thuiller, W., Georges, D., and Engler, R. (2014). *Biomod2:* *ensemble platform for species distribution modelling. R package version* 3: 1–64*.* [WWW document] URL http://CRAN.R-project.org/package=biomod2. [accessed 20 August 2018]

van Vuuren, D. P., Edmonds, J., Kainuma, M., Riahi, K., Thomson, A., Hibbard, K., Hurtt, G. C., Kram, T., Krey, V., Lamarque, J. F., Masui, T., Meinshausen, M., Nakicenovic, N., Smith, S. J., and Rose, S. K. (2011). The representative concentration pathways: an overview. *Clim. Change* 109, 5–31. doi: 10.1007/s10584-011-0148-z

Wang, S. L., Li, L., Ci, X. Q., Conran, J. G., and Li, J. (2019). Taxonomic status and distribution of *Mirabilis himalaica* (Nyctaginaceae). *J. Syst. Evol.* 57 (5), 431–439. doi: 10.1111/jse.12466

White, T. J., Bruns, T., Lee, S., and Taylor, J. (1990). “Amplification and direct sequencing of fungal ribosomal RNA genes for phylogenetics,” in *PCR protocols*: *a guide to methods and applications*, ed. M. A., Innis, D. H., Gelfand, J. J., Sninsky, and T. J., White (California: Academic Press), 315–322.

Whittall, J. B., Medina-Marino, A., Zimmer, E. A., and Hodges, S. A. (2006). Generating single-copy nuclear gene data for a recent adaptive radiation. *Mol. Phylogenet. Evol.* 39, 124–134. doi: 10.1016/j.ympev.2005.10.010

Wolf, P. G. (1995). Phylogenetic analyses of rbcL and nuclear ribosomal RNA gene sequences in Dennstaedtiaceae. *Am. Fern J.* 85, 306–327. doi: 10.2307/1547812

Wright, S. (1978). *Variability within and among natural populations, evolution and the genetics of populations*. Chicago: The University of Chicago Press.
